# Rainforest understory beetles of the Neotropics, *Mizotrechus* Bates 1872, a generic synopsis with descriptions of new species from Central America and northern South America (Coleoptera, Carabidae, Perigonini)

**DOI:** 10.3897/zookeys.145.2274

**Published:** 2011-11-04

**Authors:** Terry L. Erwin

**Affiliations:** 1 Hyper-diversity Group, Department of Entomology, MRC-187, National Museum of Natural History, Smithsonian, Institution, Washington, P.O. Box 37012, DC 20013-7012, USA

**Keywords:** Flight Intercept Traps (FITs), light traps, Neotropics, Nicaragua, Costa Rica, Panamá, Colombia, Venezuela, Guyane, Brazil

## Abstract

Information on the single previously described species, *Mizotrechus novemstriatus* Bates 1872 (type locality: Brazil – Amazonas, Tefé), is updated and 17 new species for the genus from Nicaragua, Costa Rica, Panamá, Colombia, Venezuela, and Guyane are described. The species records in the literature and on determined specimens in some collections of *Mizotrechus novemstriatus* Bates from Central America are not that species; currently, *Mizotrechus novemstriatus* is known only from its type locality in Amazonian Brazil. For the new species described, their known general distributions are as follows: *Mizotrechus batesi* **sp. n.** (Guyane), *Mizotrechus bellorum* **sp. n.** (Guyane), *Mizotrechus brulei* **sp. n.** (Guyane), *Mizotrechus belevedere* **sp. n.** (Guyane), *Mizotrechus costaricensis* **sp. n.** (Costa Rica), *Mizotrechus dalensi* **sp. n. (**Guyane), *Mizotrechus edithpiafae* **sp. n.** (provenance unknown), *Mizotrechus fortunensis* **sp. n.** (Panamá), *Mizotrechus gorgona*. **sp. n.** (Colombia), *Mizotrechus grossus* **sp. n.** (Guyane), *Mizotrechus jefe* **sp. n.** (Panamá), *Mizotrechus marielaforetae* **sp. n.** (Guyane), *Mizotrechus minutus* **sp. n.** (Guyane), *Mizotrechus neblinensis* **sp. n.** (Guyane, Venezuela), *Mizotrechus poirieri* **sp. n.** (Guyane), and *Mizotrechus woldai* **sp. n.** (Panamá). Long-term use of flight intercept traps in Guyane provided so many new species that apparently the use of FITs is the way to collect adults of this taxon, previously known from very few specimens. Many more species of this genus can be expected to be discovered throughout the Neotropics; the present contribution is a preliminary synopsis with identification key and adult images of all known species. Likely numerous species are yet to be discovered throughout tropical climes.

## Abstract (es)

Se actualiza la información sobre la única especie descrita anteriormente, *Mizotrechus novemstriatus* Bates 1872 (localidad tipo: Brasil – Amazonas, Tefé) y se describen 17 nuevas especies dentro del género provenientes de Nicaragua, Costa Rica, Panamá, Colombia, Venezuela y Guayana Francesa. Los registros para la especie en la literatura y en especímenes identificados en algunas colecciones de *Mizotrechus novemstriatus* Bates de Centroamérica no son esa especie; actualmente, *Mizotrechus novemstriatus* se conoce solamente de su localidad tipo en el área amazónica de Brasil. Para las nuevas especies que se describen, sus distribuciones conocidas son las siguientes: *Mizotrechus batesi* sp. n. (Guayana Francesa), *Mizotrechus bellorum* sp. n. (Guayana Francesa), *Mizotrechus brulei* sp. n. (Guayana Francesa), *Mizotrechus belvedere* sp. n. (Guayana Francesa), *Mizotrechus costaricensis* sp. n. (Costa Rica), *Mizotrechus dalensi* sp. n. (Guayana Francesa), *Mizotrechus edithpiafae* sp. n. (procedencia desconocida), *Mizotrechus fortunensis* sp. n. (Panamá), *Mizotrechus gorgona*. sp. n. (Colombia), *Mizotrechus grossus* sp. n. (Guayana Francesa*), Mizotrechus jefe* sp. n. (Panamá), *Mizotrechus marielaforetae* sp. n. (Guayana Francesa), *Mizotrechus minutus* sp. n. (Guayana Francesa), *Mizotrechus neblinensis* sp. n. (Guayana Francesa, Venezuela), *Mizotrechus poirieri* sp. n. (Guayana Francesa), y *Mizotrechus woldai* sp. n. (Panamá). El uso prolongado de trampas de intersección de vuelo en Guayana Francesa proveyó tantas nuevas especies que aparentemente el uso de estas trampas es la única manera para colectar adultos de este taxón, previamente conocido de muy pocos especímenes. Muchas más especies de este género se esperan descubrir a través de los Neotrópicos; esta contribución es una sinopsis preliminar con clave de identificación e imágenes de los adultos de todas las especies conocidas. Probablemente numerosas más especies están por encontrarse en los trópicos.

## Introduction

Bates (1872) proposed the genus *Mizotrechus* for several species including, *Mizotrechus novemstriatus* Bates 1872. The other species were later transferred by him (Bates, 1883) to *Perigona* Laporte de Castelnau 1835. Thus, *Mizotrechus* has been thought to be monobasic since Bates’ action in 1883. Bates (1883) also regarded a specimen from Chontales, Nicaragua collected by Janson to be the same species he described from Ega (now Tefé), Brazil. Careful study reported herein of male genitalia and body proportions indicates Bates’ determination to be incorrect. Now that many new species have emerged from Guyane (French Guiana and “Cayenne” of earlier authors) and Central American collections and I have been able to study Bates’ Ega type, I regard all Central American specimens identified as *Mizotrechus novemstriatus* Bates to be misidentifications. Given the uniformity of shape and color of species in this genus (Figs 1–18) and the previous paucity of specimens to study, it is understandable that mistakes could have been made. With the incredible new collections from Guyane, some species in ample series, it is time to set this genus on the road to a better understanding; that is the purpose of this paper which is predictably just the beginning.

## Methods and specimens

Methods and species concepts follow those previously described (Erwin and Kavanaugh 1981; Kavanaugh and Erwin 1991). The species validation and diagnosis format follows as closely as possible that suggested in Erwin and Johnson (2000). Measurements of length (ABL, SBL) and width (TW) follow those of Ball (1972) and Kavanaugh (1979): ABL (apparent body length), measured from apex of labrum to apex of longer elytron (in adults of this genus, the abdomen often protrudes beyond the elytral apex, thus the ABL often is much larger that the SBL; SBL (standardized body length), equals the sum of the lengths of the head (measured from apex of clypeus to a point on midline at level of the posterior edge of compound eyes); PL (pronotum length) is measured from apical to basal margin along midline; LE (elytron length) is measured from apex of scutellum to apex of the longer elytron; and TW (total width) measured across both elytra at their widest point with suture closed. Attributes of the elytron include the basic elytral structure occurring between the elytral intervals that frequently has been expressed as a “stria,” or “row of punctures,” etc. I refer to this structure as an interneur (Erwin 1974). Attributes of the abdominal ventral sterna are referred to using the numbering system generally accepted in Carabid studies, i.e., the sternum divided medially by the hind coxae is sternum II (the first being hidden) and the last visible is sternum VII (Liu et al. 2011). In describing attributes of the male genitalia, the proximal end of the median lobe has traditionally been referred to as the “basal bulb” in studies of Carabidae. However, in many more basal groups, the proximal end is not a scleritized bulb as it is in most higher groups such as the Lebiini. In my revision of the genus *Pericompsus* (Erwin, 1974), I encountered the same problem with the term “stria” for features of the elytra. The result was the use of the term “interneur” to apply to the attribute lying between intervals. Through use of this term, one could describe the feature as interneur striate, punctate, striatiopunctate, etc. The same holds true for the proximal end of the median lobe. In Snodgrass (1935), the term “phallobase” is used, and I have adopted it here. So, by extension, in Carabidae we can say phallobase hooded (Lebiini), phallobase of two parallel sclerotized struts (basal trechines and *Andinodontis*), phallobase of two uneven struts (*Bembidion*), etc. Kavanaugh (pers. comm.) points out that with struts there is still a connecting membrane surrounding the struts forming a “bulb.”

Included in this study are a total of 56 specimens: ten from the National Museum of Natural History, Washington, DC (NMNH) in my charge, 41 specimens received from SEAG (Société Entomologique Antilles-Guyane) in Guyane, a single specimen from INBIO collected in Costa Rica and sent to me by Angel Solis, and a single specimen from the University of Alberta, Edmonton, Canada (UASM) collected in Colombia and sent to me by George E. Ball. Also studied were the lectotype of *Mizotrechus novemstriatus* Bates from the Muséum National d‘Histoire Naturelle, Paris (MHNP, Azadeh Taghavian, Collection Manager) along with an unidentified specimen determined by Bates simply as “Mizotrechus/G^er^Bates,” and the specimen from Nicaragua that Bates identified incorrectly came from the Natural History Museum in London (BMNH, Beulah Garner, Collection Manager).

The habitus images of the adult beetles portray most of the character states referred to in the key provided. Illustrations of male genitalia are standard for descriptive taxonomy of carabid beetles in both preparation and aspects presented, as is the presentation of the female genitalia. The habitus images of the adults were made with a Visionary DigitalTM high resolution imaging system rendered using Photoshop to become “Digital Photo-illustrations.” Figure captions include an ADP number, which is a unique identification number for the specimen that was illustrated or imaged and links the specimen and associated illustrations and/or images to additional information in electronic databases at the NMNH. Representative specimens will be transferred to Guyane when they have a museum in which to keep them secure.

**Figure 39.**
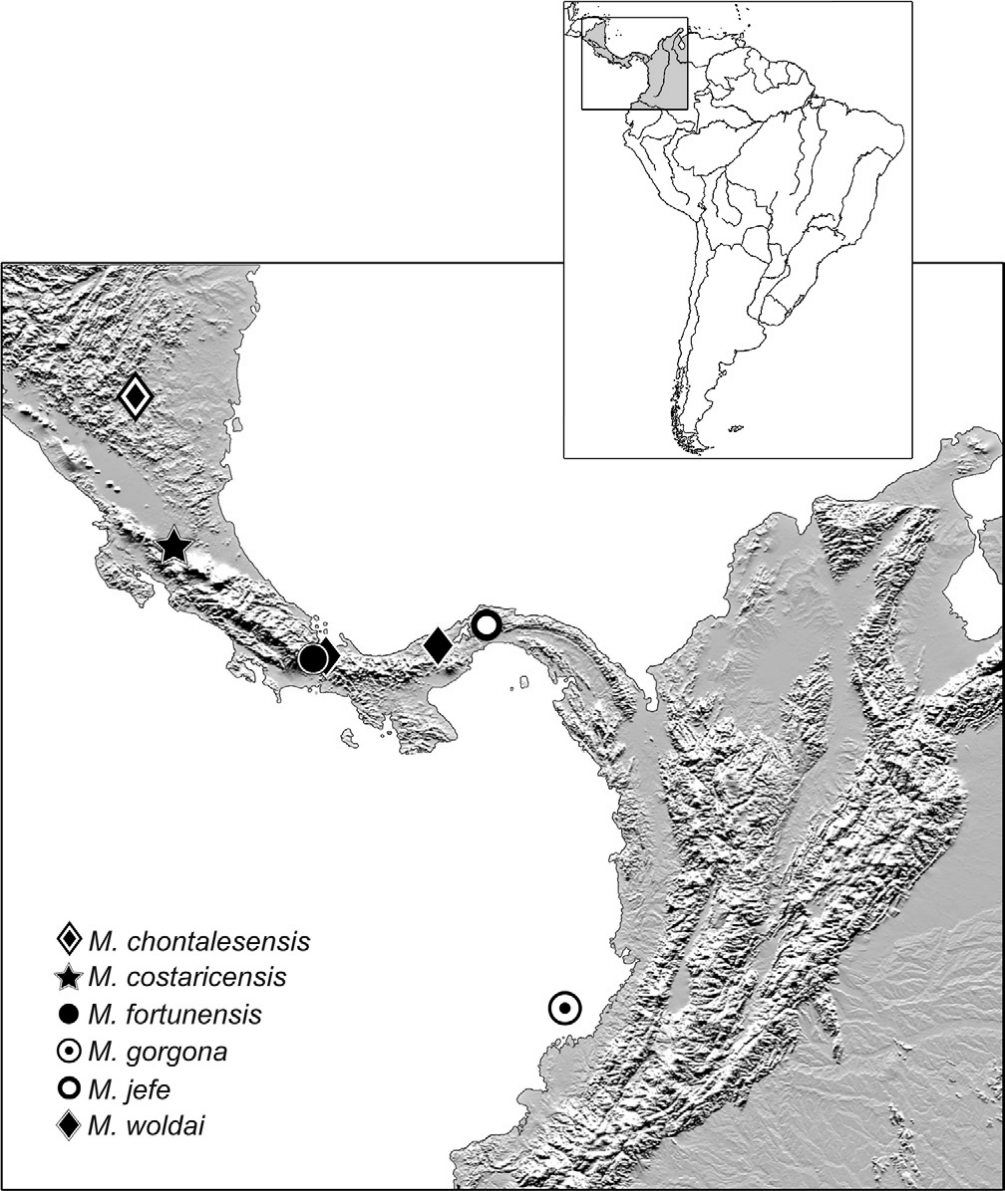
Distribution symbol map for known localities of *Mizotrechus chontalesensis*, *Mizotrechus costaricensis*, *Mizotrechus fortunensis*, *Mizotrechus gorgona*, *Mizotrechus jefe*, *Mizotrechus woldai*, spp. n.

**Figure 40.**
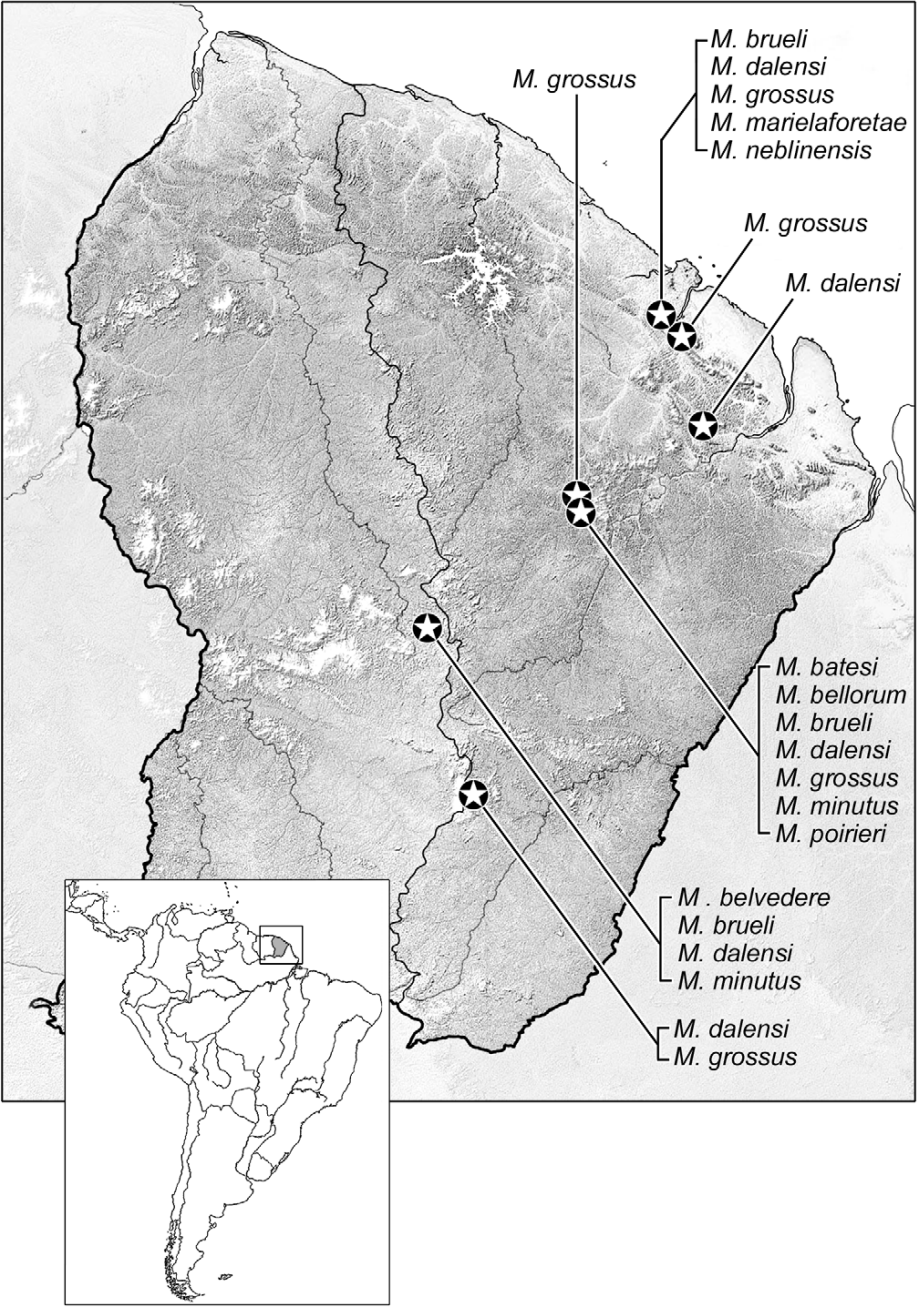
Distribution symbol map for known localities of *Mizotrechus batesi*, *Mizotrechus bellorum*, *Mizotrechus belvedere*, *Mizotrechus brulei*, *Mizotrechus dalensi*, *Mizotrechus grossus*, *Mizotrechus marielaforetae*, *Mizotrechus minutus*, *Mizotrechus poirieri*, *Mizotrechus neblinensis*, spp. n.

**Figure 41.**
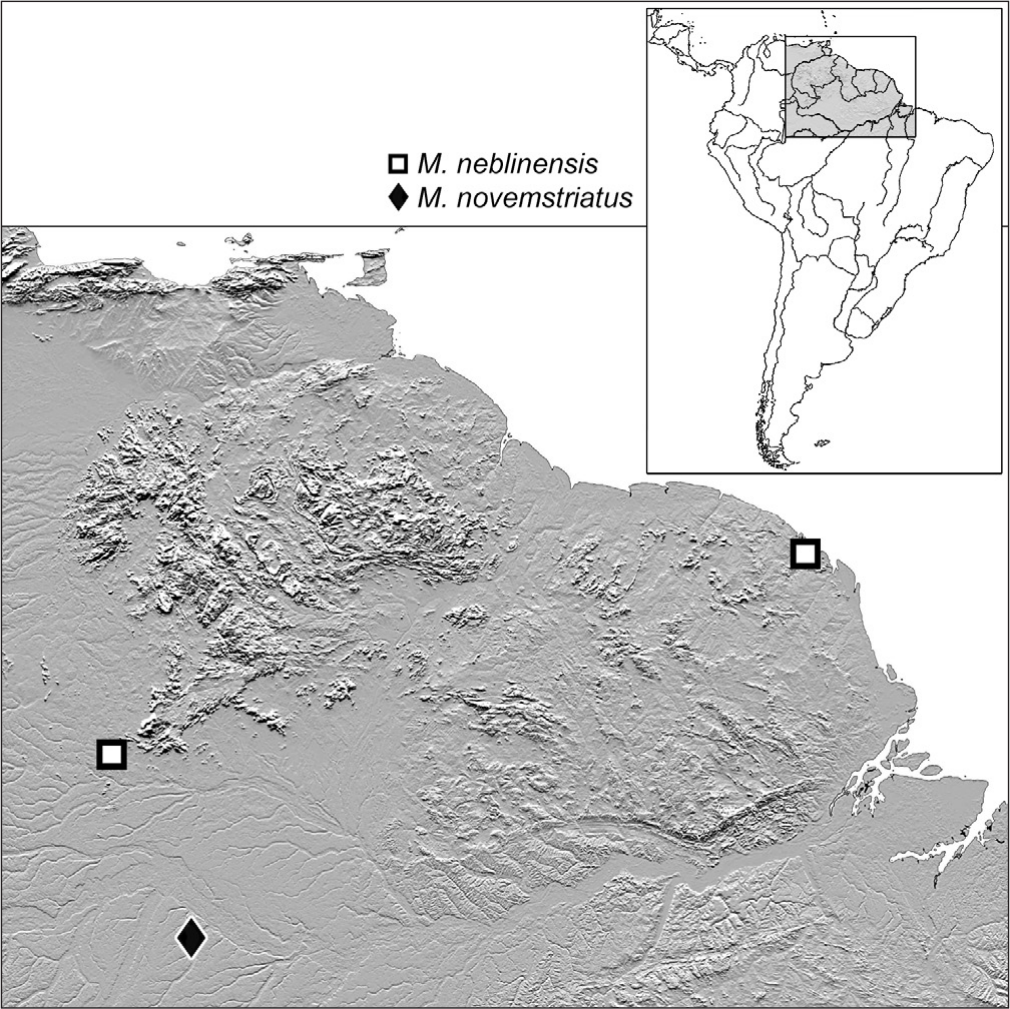
Distribution symbol map for known localities of *Mizotrechus neblinensis*, sp. n., *Mizotrechus novemstriatus* Bates.

Geographical data are presented for species based on all known specimens available at the time of manuscript preparation, including those in the literature. Georeferences have been determined from locality information provided on specimen labels; only those exact georeferences reported in decimal degrees that are provided on the label are placed in quotes, otherwise I have estimated these as closely as possible from places, mileage, etc., listed on the label and searched with Google Earth. Latitude and longitude are reported in decimal degrees. Distribution maps are provided for the species (Figs 39–41). Here, vernacular names in English are proposed, as common names are becoming increasingly needed in conservation and/or agricultural and forestry applications. These names are based on criteria set forth in Erwin (2011b) and applied in Erwin (2011a).

## Taxonomy

### Trough beetles Mizotrechus

Bates 1872

http://species-id.net/wiki/Mizotrechus

Mizotrechus Bates, 1872:199

#### Type species.

*Mizotrechus novemstriatus* Bates, 1872:199

#### Number of species.

Previously one, now 18.

#### Taxonomy.

Stable herein as of this publication, yet many new species expected in Central and South America, particularly south of the main Amazon channel. Adelphotaxon: probably *Perigona* Laporte de Castelnau 1835.

#### Proposed English vernacular name.

 Trough beetles. An attribute of adults of the Tribe Perigonini is the peculiar setiferous trough-like margin at the latero-apical portion of the elytron. While Bates’ use of “*mizo*” as part of the genus name is unclear, I found that the French translation of the Japanese word, *mizo*, is ditch or pit in English. Perhaps Bates (who published much on Japanese beetles) was referring to the long marginal setiferous “trough” near the apex of the elytron.

#### Distribution.

Presently known from Central and (northern) South America and here predicted to be found throughout the Amazon Basin and possibly north into México.

#### Habitat.

Tropical rainforest, dry forest, and cloud forest possibly in leaf litter, or more likely subcortical (under loose bark). I suggest that these beetles are subcortical based on their coloration, robust exoskeleton, markedly expanded distal antennomeres, prolonged sharp mandibles, serrate or undulating humerus in some species, and somewhat depressed form. Some of these attributes are found in the adults of genera *Pachyteles*, *Holmalomorpha*, *Catapiesis*, *Inpa*, and *Morion*, among others, whose adult members are known to be subcortical inhabitants for part of their life history.

#### References.

(Bates (1872, 1883)

#### Diagnostic combination.

 Differing in adult attributes from those of its probable adelphotaxon, *Perigona* Laporte de Castelnau 1835 and from *Diploharpus* Chaudoir 1850 by the following: 1) adult *Mizotrechus* possess markedly expanded distal antennomeres (neither adults of *Perigona* nor *Diploharpus* adults have such); 2) both male and female *Mizotrechus* adults have the foreleg femur modified in some manner (except *Mizotrechus batesi*, sp. n.) such as dentate, serrate, swollen or ridged (lacking in adults of both *Perigona* and *Diploharpus*); 3) adult *Mizotrechus* lack discal setae of the elytra (present in *Perigona* at apex of interneur 5 and in *Diploharpus* at middle and apical third in interneur 3); 4) adult *Mizotrechus* have umbilicate setae at apical third off-set and surrounded by a dense patch of short setae (both *Perigona* and *Diploharpus* have the umbilicate setae in-line and with few if any short setae); 5) adult *Mizotrechus* have a dense patch of setae in the apical trough of the elytron (neither *Perigona* nor *Diploharpus* have such a patch, although some species have a few setae); 6) sternum III of both sexes of *Mizotrechus* species have a short row of reclinate setae medially set between two narrowly spaced ambulatory setae (neither adults of *Perigona* nor *Diploharpus* nor a new genus (Erwin, in prep) have such).

#### Distribution.

Lowland tropical rainforests in Central and northern South America, as well as midland cloud and dry forests in Central America.

#### Included species.

The species list below, as well as arrangement of descriptions that follow is ordered alphabetically.

*Mizotrechus batesi* sp. n.Guyane

*Mizotrechus bellorum* sp. n.Guyane

*Mizotrechus belvedere* sp. n.Guyane

*Mizotrechus brulei* sp. n.Guyane

*Mizotrechus chontalesensis* sp. n. Nicaragua

*Mizotrechus costaricensis* sp. n.Costa Rica

*Mizotrechus dalensi* sp. n.Guyane

*Mizotrechus edithpiafae* sp. n.?

*Mizotrechus fortunensis* sp. n.Panamá

*Mizotrechus gorgona*. sp. n.Colombia

*Mizotrechus grossus* sp. n.Guyane

*Mizotrechus jefe* sp. n.Panamá

*Mizotrechus marielaforetae* sp. n.Guyane

*Mizotrechus minutus* sp. n.Guyane

*Mizotrechus neblinensis* sp. n.Guyane, Venezuela

*Mizotrechus novemstriatus* Bates 1872 Brazil

*Mizotrechus poirieri* sp. n.Guyane

*Mizotrechus woldai* sp. n.Panamá

#### Key to the species of *Mizotrechus* Bates 1872

**Table:** 

1	Elytron with striatopunctate interneurs, punctures small, evident, regularly spaced. Foreleg femur (Fig. 19) unmodified posteriorly. Size very small (ABL = 4.4–4.6 mm)	*Mizotrechus minutus*sp. n.
1’	Elytron with smooth or uneven interneurs, punctures if evident not regularly spaced. Foreleg femur modified posteriorly, either slightly swollen, ridged, dentate, or serrate. Size small to large (ABL = 5.9–8.9 mm)	2
2(1’)	Form narrow, parallel-sided (Fig. 1), pronotum nearly square; size small (ABL = 5.9 mm, narrow body)	*Mizotrechus batesi* sp. n.
2’	Form broad, pronotum sub-cordiform; size larger (ABL = 5.9 (broad body) – 8.9 mm)	3
3(2’)	Elytral margin behind humerus evidently serrate, the narrowly explanate margin dentate with minute seta at point of each tooth	4
3’	Elytral margin behind humerus not evidently serrate, smooth or rough, if rough explanate margin interrupted by minute setae and interval between setae blunt	8
4(3)	Elytron with all interneurs deep and evenly impressed throughout. Foreleg femur (Fig. 23) slightly swollen along base of postero-ventral margin in form of long ridge	*Mizotrechus brulei* sp. n.
4’	Elytron with discal interneurs moderately deep, lateral interneurs much less impressed. Foreleg femur (as in Fig. 24) serrate along basal third to half of postero-ventral margin	5
5(4’)	Size larger (ABL = 8.4 mm)	6
5’	Size smaller (ABL = 6.6–7.4 mm)	7
6(5)	Pronotum markedly convex; lateral margin straight before hind angle, a small acute tooth behind posterior lateral seta (ABL = 8.4 mm)	*Mizotrechus gorgona* sp. n.
6’	Pronotum barely convex; lateral margin sinuate before right hind angle, (ABL = 8.4 mm)	*Mizotrechus costaricensis* sp. n.
7(5’)	Pronotum barely convex, narrow; hind angle about right (ABL = 6.6 mm)	*Mizotrechus jefe* sp. n.
7’	Pronotum markedly convex, broad; hind angle a small tooth (ABL = 7.4 mm)	*Mizotrechus woldai* sp. n.
8(3’)	Elytron with all interneurs deep and evenly impressed throughout	9
8’	Elytron with discal interneurs moderately deep, lateral interneurs much less impressed	12
9(8)	Size large (ABL = 8.4 mm). Pronotum markedly convex, large with markedly arcuate side margins. Foreleg femur (Fig. 22) dentate on postero-ventral margin	*Mizotrechus edithpiafae* sp. n.
9’	Size smaller (ABL 6.5–7.1 mm). Pronotum barely convex, longer than wide, nearly quadrate. Foreleg femur (as in Fig. 21) with a short ridged on postero-ventral margin	10
10(9’)	Pronotum with hind angle sharp, about a right angle, not produced as a small denticle	*Mizotrechus chontalesensis* sp. n.
10’	Pronotum with hind angle sharp, produced as a small denticle	11
11(10’)	Pronotum nearly quadrate (W/L = 1.356)	*Mizotrechus novemstriatus* Bates
11’	Pronotum much wider than long (W/L = 1.996–2.228)	*Mizotrechus neblinensis* sp. n.
12(8’)	Pronotum with posterior lateral margin moderately lobed just anterior to dentiform hind angle	*Mizotrechus belvedere* sp. n.
12’	Pronotum with posterior lateral margin straight just anterior to dentiform hind angle or right hind angle	13
13(12’)	Size large (ABL 7.6–9.4 mm)	14
13’	Size smaller (ABL 6.0–6.8 mm)	16
14(13)	Form elongate and narrow. Elytron with margin behind humerus rough, undulating between microsetae. Foreleg femur (as in Fig. 24) serrate on postero-ventral margin	*Mizotrechus fortunensis* sp. n.
14’	Form broader and shorter. Elytron with margin behind humerus smooth, not undulating between microsetae. Foreleg femur (Fig. 20) dentate on postero-ventral margin	15
15(14’)	Elytra very convex, at least interneurs 1–3 deep and complete. Pronotum with margin anterior to hind angle slightly arcuate	*Mizotrechus dalensi* n. sp
15’	Elytra rather depressed, only interneur 1 deep and complete. Pronotum with margin anterior to hind angle straight	*Mizotrechus grossus* sp. n.
16(13’)	Pronotum with margin anterior to hind angle slightly arcuate	*Mizotrechus poirieri* sp. n.
16’	Pronotum with margin anterior to hind angle straight	17
17(16’)	Form elongate and narrow. Elytron with at least 4 interneurs deep and complete, 2–4 about equal to 1 in depth. Foreleg femur (as in Fig. 21) ridged on postero-ventral margin	*Mizotrechus marielaforetae* sp. n.
17’	Form short and broad. Elytron with only interneur 1 deep and complete, 2–4 much shallower. Foreleg femur (as in Fig. 22) dentate on postero-ventral margin	*Mizotrechus bellorum* sp. n.

## Accounts of taxa

### Bates’ trough beetle Mizotrechus batesi sp. n.

urn:lsid:zoobank.org:act:897D9EB5-D50F-4021-9818-A79D5C86175A

http://species-id.net/wiki/Mizotrechus_batesi

Figs 1 40

#### Holotype.

Guyane, Saut Pararé, Arataie River, Nouragues Field Station, 51 m, 4.0378°N, 52.6725°W, 30 November 2009 (S Brule, PH Dalens, & E Poirier)(NMNH: ADP124884, male).

#### Derivation of specific epithet.

 The epithet “*batesi*” is an eponym, based on the family name of Henry Walter Bates, whose eleven years of collecting beetles in South America capturing adults of many new species, and genera such as this, would qualify him for the Hall of Fame of beetle collectors and describers, if there was such a Hall.

#### Proposed English vernacular name.

 Bates’ trough beetle.

#### Diagnosis.

With the attributes of the genus as described above and small sized for the genus as it is presently understood; adults have castaneous integument, except anterior parts of mandible, baso-lateral corner of labrum, and clypeal suture piceous. Frons shallowly rugose and punctulate. Occiput punctulate. Pronotum quadrate with lateral margin shallowly emarginate just anterior to hind angle; base densely punctulate. Elytra narrow and elongate, about the width of pronotum across anterior third, and with 8 well-impressed interneurs, intervals flat; margin behind humerus shallowly serrulate. Foreleg femur with slightly produced ridge on postero-ventral margin.

#### Description.

(Fig. 1). *Size*: See Appendix 1. Small for genus, ABL = 5.9 mm, SBL = 4.9 mm, TW = 1.8 mm. *Color*: see diagnosis, above. *Luster*: Head, pronotum and legs shiny. *Head*: Labrum quadrate and medially notched apically. Eye small, moderately convex. Gena long, straight. Frons, occiput and gena glabrous. *Prothorax*: Broad, narrowed slightly toward base, margin beaded, not explanate except at hind angle; surface punctulate, punctures widespread, glabrous. *Pterothorax*: Elytron moderately convex, intervals slightly costate, interneurs striate, not punctate, apex slightly oblique and slightly rounded, sutural apex narrowly truncate. Metasternum sparsely setiferous in male. *Legs*: Normal in male; foreleg femur (as in Fig. 21) with slightly produced long ridge on postero-ventral margin and with a very short secondary ridge basally and above end of long ridge, not dentate; posterior trochanter tapered to point, length nearly half that of femur. *Abdomen*: Abdominal sterna moderately setiferous; sternum IV of male with narrow and dense patch of decumbent setae; sternum VII medially notched in male. *Male genitalia*: Aedeagus and parameres missing from single known male holotype, although the ring sclerite is present. *Female genitalia*: Unknown.

**Plate 1.**
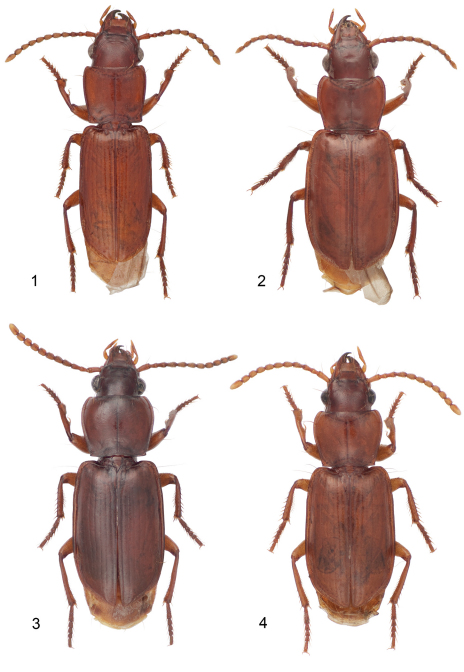
Digital Photo-illustrations, habitus, dorsal aspect: **1** *Mizotrechus batesi* sp. n.,ABL = 5.9 mm, male holotype, ADP124884; type locality. **2** *Mizotrechus bellorum* sp. n.**,** ABL = 6.9 mm, male holotype, ADP124890; type locality. **3** *Mizotrechus belvedere* sp. n.**,** ABL = 7.2 mm, male holotype, ADP129201; type locality. **4** *Mizotrechus brulei* sp. n.**,** ABL = 6.7 mm, female paratype, ADP124926; type locality.

#### Dispersal potential.

These beetles are macropterous and capable of flight. They are moderately swift and agile runners.

#### Way of life.

The adult holotype was collected in a flight intercept trap in the rainforest understory. The adult holotype was active in November, at the end of the dry season.

#### Other specimens examined.

None.

#### Geographic distribution.

(Fig. 40). This species is currently known only from the type locality in the lowlands of Guyane.

### Bells’ trough beetle Mizotrechus bellorum sp. n.

urn:lsid:zoobank.org:act:E336EDFB-B152-4B87-8C8E-1AE5A4B8B377

http://species-id.net/wiki/Mizotrechus_bellorum

Figs 2 25 40

#### Holotype.

Guyane, Saut Pararé, Arataie River, Nouragues Field Station, 51 m, 4.0378°N, 52.6725°W, 13 September 2009 (S Brule, PH Dalens, & E Poirier)(NMNH: ADP124890, male)

#### Derivation of specific epithet. 

The epithet “*bellorum*” is an eponym, based on the family name of Ross and Joyce Bell, with a special thanks for a career that brought light to the rhysodine clade of very interesting beetles with colors and tough cuticle also found in adults of *Mizotrechus* species.

#### Proposed English vernacular name.

 Bells’ trough beetle.

#### Diagnosis.

With the attributes of the genus as described above and medium sized for the genus as it is presently understood; adults have castaneous integument, except anterior parts of mandible, baso-lateral corner of labrum, and clypeal suture piceous. Frons at sides with evident rugae, punctulate. Occiput at sides with evident rugae, punctulate. Pronotum nearly quadrate, narrowed behind, with straight margins to hind angle, hind angle dentate, tooth small; base sparsely rugulose. Elytra broad and short, much wider than the width of pronotum across anterior third, and with only interneurs 1–3 moderately engraved, 4–8 evident yet shallowly impressed, more so toward margin, not punctate; margin behind humerus uneven, yet not serrate. Foreleg femur subdentate at the base of postero-ventral margin.

#### Description.

(Figs 2, 25). *Size*: See Appendix 1. Medium sized for the genus, ABL = 6.9 mm, SBL = 5.87 mm, TW = 2.55 mm. *Color*: see diagnosis, above. *Luster*: Head, pronotum, and legs shiny, elytra slightly duller due to shallowly engraved slightly stretched microsculpture. *Head*: Labrum quadrate and apico-medially emarginate. Eye large, moderately convex. Gena moderately short, straight. Frons, occiput and gena glabrous. *Prothorax*: Narrow, quadrate, narrowed slightly toward base, margins not emarginate before hind angle, angle dentate, tooth small, margin moderately explanate except wider at hind angle; surface punctulate, punctures widespread, glabrous. *Pterothorax*: Elytron moderately convex, disk flat, intervals flat, interneurs not punctate, apex moderately oblique and straight, sutural apex narrowly rounded. Metasternum sparsely setiferous in male. *Legs*: Normal in male; foreleg femur (as in Fig. 20) with a small obtuse tooth at basal third of postero-ventral margin; posterior trochanter narrowly acute at apex, about half the length of the femur. *Abdomen*: Abdominal sterna moderately setiferous; sternum IV of male with narrow and dense patch of decumbent setae. *Male genitalia*: Median lobe (Fig. 25) elongate and robust with ostium moderately elongate, over half the length of the median lobe; apex a losp. n.tulate distal end less bent ventrad than in *Mizotrechus dalensi* and thicker in cross section, moderately curved in lateral aspect, ventral margin proximal to apex straight then evenly curved to apex; endophallus with complexly folded tracheal fields; phallobase hooded, not crested, opening more or less 30 degrees off axis with that of shaft. Parameres large, left a half longer than the right, both broadly rounded, asetose. *Female genitalia*: Unknown.

#### Dispersal potential.

These beetles are macropterous and capable of flight. They are moderately swift and agile runners.

#### Way of life.

The adult holotype was collected in a flight intercept trap in the rainforest understory. Adults are active in September, near the end of the dry season.

#### Other specimens examined.

 None.

#### Geographic distribution.

 (Fig. 40). This species is currently known only from the type locality in the lowlands of Guyane.

### Brule’s trough beetle Mizotrechus belvedere sp. n.

urn:lsid:zoobank.org:act:8239C95E-35C9-450D-93A0-6FF8B48ED39F

http://species-id.net/wiki/Mizotrechus_belvedere

Figs 3 26 40

#### Holotype.

 GUYANE, Saül, Commune de Saül, Belvédère de Saül, 283–325 m, 3.6223°N, 53.2159°W, 17 February 2010 (S Brule, PH Dalens, & E Poirier)(NMNH: ADP129201, male).

#### Derivation of specific epithet.

 The epithet “*belvedere*” is a singular Latinized masculine noun in apposition, based on the name of the area in which these beetles are found.

#### Proposed English vernacular name.

 Belvédère trough beetle.

#### Diagnosis.

With the attributes of the genus as described above and moderately large sized for the genus as it is presently understood; adults have castaneous integument, except anterior parts of mandible, baso-lateral corner of labrum, and clypeal suture piceous. Frons shallowly rugose above and behind eye in an arc and moderately punctulate. Occiput moderately punctulate. Pronotum longer than wide with lateral margins moderately explanate and basally notched; base moderately microrugose. Elytra moderately broad and short, apex prolonged, wider than the width of pronotum across anterior third, and with 5 well-impressed irregularly punctulate interneurs, intervals not convex; margin behind humerus rough, intervals between microsetae blunt. Foreleg femur with slightly produced postero-ventral margin.

#### Description.

(Figs 3, 26). *Size*: See Appendix 1. Moderately small for genus, ABL = 7.2 mm, SBL = 6.14 mm, TW = 2.48 mm. *Color*: see diagnosis, above. *Luster*: Head, pronotum and legs shiny, elytra matte. *Head*: Labrum quadrate, apico-medially slightly V-notched. Eye moderately large and convex. Gena with very slight bulge. Frons, occiput and gena glabrous. *Prothorax*: Moderately narrow, narrowed slightly toward base, margin moderately explanate, wider before hind angle; surface punctulate, punctures moderately dense, glabrous. *Pterothorax*: Elytron barely convex, intervals nearly flat, interneurs with well-impressed irregularly-spaced punctulate, apex slightly obliquely prolonged and slightly rounded at extreme sutural apex. Metasternum sparsely setiferous in male. *Legs*: Normal in male; foreleg femur (as in Fig. 23) with slightly produced ridge along postero-ventral margin, not dentate, without short dorsal ridge at base of longer ridge; posterior trochanter tapered to rounded point in male, length half that of femur. *Abdomen*: Abdominal sterna moderately setiferous, densely so medially on II and III; sternum III of male with short dense patch of decumbent setae medially set between two ambulatory setae; sternum VII shallowly and medially notched in male. *Male genitalia*: Median lobe (Fig. 26) short and robust with ostium moderately elongate, over half the length of the median lobe; apex a short blunt distal end that is less prominent than in *Mizotrechus brulei*, slightly curved in lateral aspect, ventral margin proximal to apex evenly curved; endophallus with complexly folded tracheal fields; phallobase hooded, opening more or less 20 degrees off axis of shaft. Parameres large, left a third longer than the right, both broadly rounded, asetose. *Female genitalia*: Unknown.

#### Dispersal potential.

These beetles are macropterous and capable of flight. They are moderately swift and agile runners.

#### Way of life.

The adult holotype was collected in a flight intercept trap in the rainforest understory. The holotype was active in February, the rainy season.

#### Other specimens examined.

 None.

#### Geographic distribution.

 (Fig. 40). This species is currently known only from the type locality in the lowlands of Guyane.

### Brule’s trough beetle Mizotrechus brulei sp. n.

urn:lsid:zoobank.org:act:59CE9DC7-C3BF-4DAE-9CE1-D72FF093AAA6

http://species-id.net/wiki/Mizotrechus_brulei

Figs 4 27 40

#### Holotype.

GUYANE, Saut Pararé, Arataie River, Nouragues Field Station, 51 m, 4.0378°N, 52.6725°W, 13 September 2009 (S Brule, PH Dalens, & E Poirier)(NMNH: ADP124886, male).

#### Derivation of specific epithet.

 The epithet “*brulei*” is an eponym, based on the family name of Stephané Brule, whose team in Guyane has been collecting beetles using Flight Intercept Traps and capturing adults of many new species, such as this one.

#### Proposed English vernacular name.

 Brule’s trough beetle.

#### Diagnosis.

With the attributes of the genus as described above and small sized for the genus as it is presently understood; adults have castaneous integument, except anterior parts of mandible, baso-lateral corner of labrum, and clypeal suture piceous. Frons shallowly rugose only at extreme anterior angles and punctulate. Occiput punctulate. Pronotum quadrate with lateral margins basally shallowly emarginate; base moderately microrugose. Elytra moderately narrow and elongate, wider than the width of pronotum across anterior third, and with 8 well-impressed interneurs, intervals not convex; margin behind humerus moderately serrulate. Foreleg femur with slight swelling near the base on the postero-ventral margin.

#### Description.

(Figs 4, 27). *Size*: See Appendix 1. Moderately small for genus, ABL = 6.0–6.8 mm, SBL = 5.53–6.41 mm, TW = 2.03–2.22 mm. *Color*: see diagnosis, above. *Luster*: Head, pronotum and legs shiny. *Head*: Labrum quadrate, apico-medially barely emarginate. Eye small, moderately convex. Gena long, straight. Frons, occiput, and gena glabrous. *Prothorax*: Broad, narrowed slightly toward base, margin beaded, not explanate except at hind angle; surface punctulate, punctures widespread, glabrous. *Pterothorax*: Elytron barely convex, intervals nearly flat, interneurs striate, not punctate, apex slightly oblique and slightly rounded, sutural apex narrowly truncate. Metasternum sparsely setiferous in male. *Legs*: Normal in both sexes; foreleg femur (Fig. 23) with slightly produced ridge along postero-ventral margin, not dentate, without short dorsal ridge at base of longer ridge; posterior trochanter tapered to acute point in male, acuminate in female, length half that of femur. *Abdomen*: Abdominal sterna moderately setiferous; sternum IV of male with narrow and dense patch of decumbent setae; sternum VII medially notched in male, slightly emarginated in female. *Male genitalia*: Median lobe (Fig. 27) short and robust with ostium moderately elongate, over half the length of the median lobe; apex short, blunt, distal end more prominent than in *Mizotrechus belvedere*, slightly curved in lateral aspect, ventral margin proximal to apex evenly curved; endophallus with complexly folded tracheal fields; phallobase hooded, opening more or less in line with axis of shaft. Parameres large, left a third longer than the right, both broadly rounded, asetose. *Female genitalia*: Not investigated; however, it is likely similar to that illustrated on Plate 11.

#### Dispersal potential.

These beetles are macropterous and capable of flight. They are moderately swift and agile runners.

#### Way of life.

The adult specimens were collected in flight intercept traps in the rainforest understory. Adults are active in April and September, in both the rainy and dry seasons.

#### Other specimens examined.

**Paratypes**: GUYANE, Montagne des Chevaux, Commune de Roura, RN2 PK22, 90 m, 4.7127°N, 52.3966°W, 14 April 2010 (S Brule, PH Dalens, & E Poirier)(NMNH: ADP124960, female), 9 January 2011 (NMNH: ADP124924, female), 6 March 2010 (NMNH: ADP127159, male), 8 August 2010 (NMNH: ADP129205, male), 22 August 2010 (NMNH: ADP128729, male, ADP128727, female), 3 October 2010 (NMNH: ADP128731, male), Saül, Commune de Saül, Belvédère de Saül, 283–325 m, 3.6223°N, 53.2159°W, 29 October 2010 (S Brule, PH Dalens, & E Poirier)(NMNH: ADP124926, female).

#### Geographic distribution.

 (Fig. 40). This species is currently known only from three lowland localities in Guyane.

### Chontales trough beetle Mizotrechus chontalesensis sp. n.

urn:lsid:zoobank.org:act:638541F2-014B-45D7-8A7E-C0D80817DEA5

http://species-id.net/wiki/Mizotrechus_chontalesensis

Figs 5 21 39

#### Holotype.

 NICARAGUA, Chontales, ? nr. Santo Domingo, 514 m, 12.262°N, 85.083°W, (EM Janson)(BMNH: ADP127181, female).

#### Derivation of specific epithet

. The epithet “*chontalesensis*” is a Latinized noun in apposition, based on the name of the area in which these beetles are found.

#### Proposed English vernacular name.

 Chontales trough beetle.

#### Diagnosis.

With the attributes of the genus as described above and moderately large sized for the genus as it is presently understood; adults have castaneous integument, except anterior parts of mandible, baso-lateral corner of labrum, and clypeal suture piceous. Frons and occiput quite smooth, without regular rugae; micropunctation widespread and hardly evident. Pronotum longer than wide with lateral margins narrowly explanate and basally shallowly emarginate with sharp, not denticulate, hind angles; base smooth, without regular microrugosity. Elytra moderately broad and short, apices not prolonged, wider than the width of pronotum across anterior third, and each with 8 well-impressed irregularly punctulate interneurs, intervals not convex; margins behind humeri rough, intervals between microsetae blunt. Foreleg femur with slightly produced ridge on postero-ventral margin.

#### Description.

(Figs 5, 21). *Size*: See Appendix 1. Moderately sized for genus, ABL = 7.1 mm, SBL = 5.87 mm, TW = 2.29 mm. *Color*: see diagnosis, above. *Luster*: Head, pronotum, elytra, and legs shiny. *Head*: Labrum quadrate, apico-medially barely emarginate. Eye small and barely convex. Gena straight. Frons, occiput, and gena glabrous. *Prothorax*: Moderately narrow, narrowed slightly toward base, margin narrowly explanate, slightly wider before hind angle; surface punctulate, punctures widespread, very fine, glabrous. *Pterothorax*: Elytron barely convex, intervals nearly flat, all interneurs with well-impressed irregularly-spaced punctures, apex not prolonged, slightly rounded at extreme sutural apex. *Legs*: Normal in female; foreleg femur (Fig. 21) with slightly produced, short, arcuate ridge on postero-ventral margin at basal forth, not dentate; posterior trochanter tapered to rounded point in female, length half that of femur. *Abdomen*: Abdominal sterna moderately setiferous, densely so medially on II and III; sternum VII barely medially emarginate in female. *Male genitalia*: Unknown. *Female genitalia*: Not investigated; however, it is likely similar to that illustrated on Plate 11.

**Plate 2.**
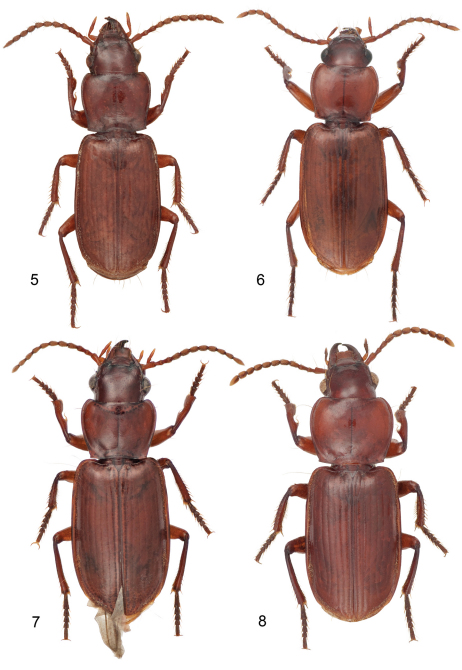
Digital Photo-illustrations, habitus, dorsal aspect: **5** *Mizotrechus chontalesensis* sp. n.**,** ABL = 7.1 mm, female holotype, ADP127181; type locality. **6** *Mizotrechus costaricensis* sp. n.**,** ABL = 8.4 mm, male holotype, ADP128620; type locality. **7** *Mizotrechus dalensi* sp. n**.,** ABL = 8.2 mm, male holotype, ADP124894; type locality. **8** *Mizotrechus edithpiafae* sp. n.**,** ABL = 8.4 mm, female holotype, ADP124948; locality unknown.

#### Dispersal potential.

These beetles are macropterous and capable of flight. They are moderately swift and agile runners.

#### Way of life.

Unknown.

#### Other specimens examined.

 None.

#### Geographic distribution.

 (Fig. 39). This species is currently known only from the type locality in the dry tropical forested lowlands of Nicaragua.

### Costa rica trough beetle Mizotrechus costaricensis sp. n.

urn:lsid:zoobank.org:act:FE064F4E-01E2-456D-8B59-6374BDECCF4F

http://species-id.net/wiki/Mizotrechus_costaricensis

Figs 6 28 39

#### Holotype.

 COSTA RICA, Alajuela, San Ramón, Reserva Biologica Alberto Brenes, Rio San Lorenzo, 850 m, 10.2283°N, 84.5857°W, 30 June – 5 July 1999 (J Rodriguez)(INBIO: ADP128620, INB0003057334, male).

#### Derivation of specific epithet.

 The epithet “*costaricensis*” is a Latinized noun in apposition, based on a geographic name formed from “Costa Rica,“ the country in which an adult of this species has been found, and the Latin suffix “-*ensis*,“ meaning of, or from that place.

#### Proposed English vernacular name.

 Costa Rica trough beetle.

#### Diagnosis.

With the attributes of the genus as described above and large sized for the genus as it is presently understood; adults have castaneous integument, except anterior parts of mandible, baso-lateral corner of labrum, and clypeal suture piceous. Frons and occiput shallowly rugose above and behind eye in an arc asp. n.rsely and finely punctulate. Pronotum subcordiform with lateral margins moderately explanate and basally emarginate before right hind angles; base shallowly and regularly microrugose. Elytra moderately narrow and short, apex prolonged, wider than the width of pronotum across anterior third, and with 5 well-impressed irregularly punctulate interneurs, intervals not convex; margin behind humerus rough, intervals between microsetae blunt. Foreleg femur with markedly serrate postero-ventral margin.

#### Description.

(Figs 6, 28). *Size*: See Appendix 1. Large for genus, ABL = 8.4 mm, SBL = 6.88 mm, TW = 2.88 mm. *Color*: see diagnosis, above. *Luster*: Head, pronotum and legs shiny, elytra matte. *Head*: Labrum quadrate, apico-medially slightly produced. Eye moderately small and convex. Gena straight. Frons, occiput, and gena glabrous. *Prothorax*: Moderately narrow, narrowed slightly toward base, margin narrowly explanate, wider before hind angle; surface sparsely punctulate, punctures very fine, glabrous. *Pterothorax*: Elytron barely convex, intervals nearly flat, 5 interneurs with well-impressed irregularly spaced punctures, apex not prolonged, slightly rounded at extreme sutural apex. Metasternum sparsely setiferous in male. *Legs*: Normal in male; foreleg femur (as in Fig. 24) with markedly serrate postero-ventral margin, not dentate; posterior trochanter tapered to acute point in male, length half that of femur. *Abdomen*: Abdominal sterna moderately setiferous, densely so medially on II and III; sternum III of male with short row of decumbent setae medially set between two ambulatory setae; sternum VII shallowly and medially notched in male. *Male genitalia*: Median lobe (Fig. 28) short and robust with ostium moderately elongate, over half the length of the median lobe; apex with a very short and narrow distal end, slightly rounded in lateral aspect, more twisted laterally than in *Mizotrechus fortunensis*, ventral margin proximal to apex straight then briefly curved to apex; endophallus with complexly folded tracheal fields; phallobase hooded, opening more or less 30 degrees off axis of shaft. Parameres large, left a twice longer than the right, both broadly rounded, asetose. *Female genitalia*: Unknown.

#### Dispersal potential.

These beetles are macropterous and capable of flight. They are moderately swift and agile runners.

#### Way of life.

The holotype was collected in an aerial net. Adults are active in early July, the area’s dry season.

#### Other specimens examined. 

None.

#### Geographic distribution.

 (Fig. 39). This species is currently known only from the type locality in premontane rainforests of Costa Rica.

### Dalens’ trough beetle Mizotrechus dalensi sp. n.

urn:lsid:zoobank.org:act:1FAB3B90-4F18-4690-825D-B13F8510E82E

http://species-id.net/wiki/Mizotrechus_dalensi

Figs 7 20 29 40 Plate 11

#### Holotype.

GUYANE, Saut Pararé, Arataie River, Nouragues Field Station, 51 m, 4.0378°N, 52.6725°W, 30 November 2009 (S Brule, PH Dalens, & E Poirier)(NMNH: ADP124894, male).

#### Derivation of specific epithet.

 The epithet “*dalensi*” is an eponym, based on the family name of P.H. Dalens, whose team in Guyane has been collecting beetles using Flight Intercept Traps and capturing adults of many new species, such as this one.

#### Proposed English vernacular name.

 Dalens’ trough beetle.

#### Diagnosis.

With the attributes of the genus as described above and medium sized for the genus as it is presently understood; adults have castaneous integument, except anterior parts of mandible, baso-lateral corner of labrum, and clypeal suture piceous. Frons shallowly rugose and punctulate. Occiput shallowly rugose at sides and punctulate. Pronotum quadrate with lateral margins basally straight to slightly toothed hind angle; base moderately rugose. Elytra broad and moderately short, much wider than width of pronotum across anterior third, and with two well- impressed interneurs, the remainder shallower toward margin; margins behind humeri not serrulate. Foreleg femur dentate basally on postero-ventral margin.

#### Description.

(Figs 7, 20, 29, Plate 11). *Size*: See Appendix 1. Medium-sized for genus, ABL = 7.6–8.2 mm, SBL = 6.40–7.09 mm, TW = 2.64–3.11 mm. *Color*: see diagnosis, above. *Luster*: Head, pronotum and legs shiny; elytra dull due to marked slightly stretched well-impressed microsculpture. *Head*: Labrum quadrate, apico-medially barely emarginate. Eye moderately large, moderately convex. Gena moderately long, straight. Frons, occiput, and gena glabrous. *Prothorax*: Broad, narrowed slightly toward base, margin beaded, not explanate except at hind angle; surface punctulate, punctures widespread, and with shallow yet evident transverse microsculpture, surface glabrous. *Pterothorax*: Elytron moderately convex, intervals flat, interneurs striate, not punctate, becoming shallower toward margin, apex slightly oblique, straight, sutural apex narrowly and slightly rounded. Metasternum sparsely setiferous in male. *Legs*: Normal in both sexes; foreleg femur (Fig. 20) dentate at basal third on postero-ventral margin; posterior trochanter tapered to acute point in male, slightly acuminate in female, length half that of femur. *Abdomen*: Abdominal sterna moderately setiferous; sternum IV of male with narrow and dense patch of decumbent setae; sternum VII medially V-notched in male, U-notched in female. *Male genitalia*: Median lobe (Fig. 29) elongate and robust with ostium moderately elongate, over half the length of the median lobe; apex a losp. n.tulate distal end less bent ventrad than in *Mizotrechus poirieri*, moderately curved in lateral aspect, ventral margin proximal to apex straight then evenly curved to apex; endophallus with complexly folded tracheal fields; phallobase hooded and crested, opening more or less 40 degrees off axis of shaft. Parameres large, left a third longer than the right, both broadly rounded, asetose. *Female genitalia*: (Plate 11). Ovipositor with broad laterotergite (**lt**) and two gonocoxites (**gc 1, gc 2**); gonocoxite 1 apico-laterally setose; gonocoxite 2 falcate, base (**b**) medium-size, narrow, blade (**bl**) elongate, with two dorsal ensiform setae (**des**), and one ventral ensiform seta (**ves**), all ensiform setae moderately long and robust; with ventral preapical nematiform setae (evident on A, hidden on B). Reproductive tract (Plate 11A) proximally with short, broad bursa copulatrix (**bc**), continuous at its distal end with common oviduct (**co**) and long robust spermatheca (**sp**), latter slightly narrowed distally; spermathecal gland (**sg**) bulbous; spermathecal gland duct (**sgd**) long, slender, attached to spermatheca at base of its broadened portion.

#### Dispersal potential.

These beetles are macropterous and capable of flight. They are moderately swift and agile runners.

#### Way of life.

The adults at four localities were collected in flight intercept traps in the rainforest understory. Adults are active in March and November, in both the rainy and dry seasons.

#### Other specimens examined.

**Paratypes**: Guyane, Saut Pararé, Arataie River, Nouragues Field Station, 51 m, 4.0378°N, 52.6725°W, 23 November 2009 (S Brule, PH Dalens, & E Poirier)(NMNH: ADP124896, male); Mount Itoupe, 570 m, 3.0148°N, 53.0721°W, 17 March 2010 (S Brule, PH Dalens, & E Poirier)(NMNH: ADP126485, male); Montagne des Chevaux, Commune de Roura, RN2 PK22, 90 m, 4.7127°N, 52.3966°W, 8 August 2010 (NMNH: ADP129203, female), 1 November 2009 (S Brule, PH Dalens, & E Poirier)(NMNH: ADP124956, female), Saül, Commune de Saül, Belvédère de Saül, 283–325 m, 4.7127°N, 52.3966°W, 20 December 2010 (NMNH: ADP127167, male), 4 January 2011 (S Brule, PH Dalens, & E Poirier)(NMNH: ADP124922, female), 36 km W Regina, Petite Montague Tortue, P17, 94 m, 4.3204°N, 52.2404°W, 23 June 2010 (G Lamarre)(NMNH: ADP127161, male).

#### Geographic distribution.

 (Fig. 40). This species is currently known from five localities in the lowland and midland rainforests of Guyane.

### Piaf’s trough beetle Mizotrechus edithpiafae sp. n.

urn:lsid:zoobank.org:act:6D88FB32-8C21-49DA-BEAD-719E3ABB3AB6

http://species-id.net/wiki/Mizotrechus_edithpiafae

Figs 8 22 30

#### Holotype.

Locality unknown. Specimen, (BMNH: ADP128624, male), labeled (Bates’ handwriting) “Mizotrechus / G^er^ Bates.”

#### Derivation of specific epithet.

 The epithet “*edithpiafae*” is an eponym, based on the full name of Edith Piaf, the famous French singer, 19 December 1915–11 October 1963, whose voice had an incredible range of diversity, as is that found in the carabid species richness of Guyane, and who sang a variety of “torch songs”, and here I play on the word “torch”, the same word that applies to what is used to ignite the trees of the unique tropical rainforests of South America, an Armageddon in our own times.

#### Proposed English vernacular name.

 Piaf’s trough beetle.

#### Diagnosis

**.** With the attributes of the genus as described above and large sized for the genus as it is presently understood. Adults with darkly infuscate integument, elytra slightly paler, except anterior parts of mandible, baso-lateral corner of labrum, and clypeal suture black. Frons and occiput smooth above and behind eye asp. n.rsely and finely punctulate. Pronotum quadrate and highly convex with lateral margins narrowly explanate and basally straight before slightly denticulate hind angles; base shallowly and regularly microrugose. Elytra very broad and short, apex not prolonged, wider than the width of pronotum across anterior third, and with 8 well- impressed irregularly punctulate interneurs, intervals not convex; margin behind humerus rough, intervals between microsetae blunt. Foreleg femur with markedly dentate ventral margin.

#### Description.

(Figs 8, 22). *Size*: See Appendix 1. Large for genus, ABL = 8.4 mm, SBL = 7.16 mm, TW = 2.94 mm. *Color*: see diagnosis, above. *Luster*: Head, pronotum, and legs shiny, elytra slightly matte. *Head*: Labrum quadrate, apico-medially moderately v-notched. Eye moderately small and convex. Gena straight. Frons, occiput, and gena glabrous. *Prothorax*: Broad, barely narrowed toward base, margin narrowly explanate, wider before hind angle; surface sparsely punctulate, punctures very fine, glabrous. *Pterothorax*: Elytron barely convex, intervals nearly flat, 8 interneurs with well-impressed irregularly-spaced punctures, apex not prolonged, slightly rounded at extreme sutural apex. Metasternum sparsely setiferous in male. *Legs*: Normal in male; foreleg femur (Fig. 22) ridged along the posterio-ventral margin and markedly produced at middle of ridge ; posterior trochanter tapered to rounded point in male, length half that of femur. *Abdomen*: Abdominal sterna moderately setiferous, densely so medially on II and III; sternum III of male with short dense row of decumbent setae medially set between two ambulatory setae; sternum VII shallowly and medially notched in male. *Male genitalia*: Median lobe (Fig. 30) short and robust with ostium moderately elongate, over half the length of the median lobe; apex with a moderately long narrow distal end, slightly curved in lateral aspect, ventral margin proximal to apex evenly curved, ventral margin proximal to apex straight; endophallus (everted) with complexly folded tracheal fields and a narrow sclerotized rod; phallobase not fully hooded, opening 20 degrees off axis of shaft. Parameres large, left a third longer than the right, both broadly rounded, asetose. *Female genitalia*: Unknown.

#### Dispersal potential.

These beetles are macropterous and capable of flight. They are moderately swift and agile runners.

#### Way of life.

Unknown.

#### Other specimens examined.

 None.

#### Geographic distribution.

 Unknown.

### Fortuna trough beetle Mizotrechus fortunensis sp. n.

urn:lsid:zoobank.org:act:CCCF2C24-8587-42A0-83A5-BD953EF41585

http://species-id.net/wiki/Mizotrechus_fortunensis

Figs 9 31 39

#### Holotype.

PANAMÁ, Chiriquí Province, Fortuna, 1050 m, 8.7341°N, 82.2701°W, 13 August 1978 (H. Wolda)(NMNH: ADP124968, male).

#### Derivation of specific epithet.

 The epithet “*fortunensis*” is a Latinized noun in apposition, based on a geographic name formed from “Fortuna“ (Panamá) the place in which adults of this species have been found, and the Latin suffix “-*ensis*,“ meaning of, or from that place.

#### Proposed English vernacular name.

 Fortuna trough beetle.

#### Diagnosis.

With the attributes of the genus as described above and large sized for the genus as it is presently understood; adults have castaneous integument, except anterior parts of mandible, baso-lateral corner of labrum, and clypeal suture piceous. Frons smooth, not or barely rugose near eye, surface punctulate. Occiput smooth, punctulate. Pronotum quadrate with lateral margins basally shallowly emarginated, hind angles slightly toothed; base densely microrugose. Elytra broad and short, much broader than width of pronotum across anterior third, and with 3 well-impressed interneurs, others shallower toward margin; margins behind humeri shallowly serrulate. Foreleg femur serrate on postero-ventral margin.

#### Description.

(Figs 9, 31). *Size*: See Appendix 1. Large-sized for genus, ABL = 8.5–8.7 mm, SBL = 7.02–7.66 mm, TW = 2.93 –3.03 mm. *Color*: see diagnosis, above. *Luster*: Head, pronotum, and legs shiny; elytra dull due to marked slightly stretched well-impressed microsculpture. *Head*: Labrum quadrate, entire apically. Eye moderately large, moderately convex. Gena moderately long, straight. Frons, occiput, and gena glabrous. *Prothorax*: Quadrate, narrowed slightly toward base, margin beaded, not explanate except at hind angle, hind angle feebly dentate; surface punctulate, punctures widespread, and with microsculpture nearly effaced, surface glabrous. *Pterothorax*: Elytron moderately convex, disk flat, intervals flat, interneurs striate, not punctate, shallower toward margin, apex slightly oblique, straight, sutural apex narrowly and slightly rounded. Metasternum sparsely setiferous in male. *Legs*: Normal in both sexes; foreleg femur (as in Fig. 24) serrate on postero-ventral margin; posterior trochanter narrowly rounded in both sexes, length half that of femur. *Abdomen*: Abdominal sterna moderately setiferous; sternum IV of male without medial patch of decumbent setae; sternum VII medially and shallowly V-notched in male. *Male genitalia*: Median lobe (Fig. 31) short and robust with ostium moderately elongate, over half the length of the median lobe; apex with a very short and narrow distal end, slightly rounded in lateral aspect, less twisted laterally than in *Mizotrechus costaricensis*, ventral margin proximal to apex straight then briefly curved to apex; endophallus with complexly folded tracheal fields; phallobase hooded, opening more or less 20 degrees off axis of shaft. Parameres large, left twice longer than the right, both broadly rounded, asetose. *Female genitalia*: Unknown.

**Plate 3.**
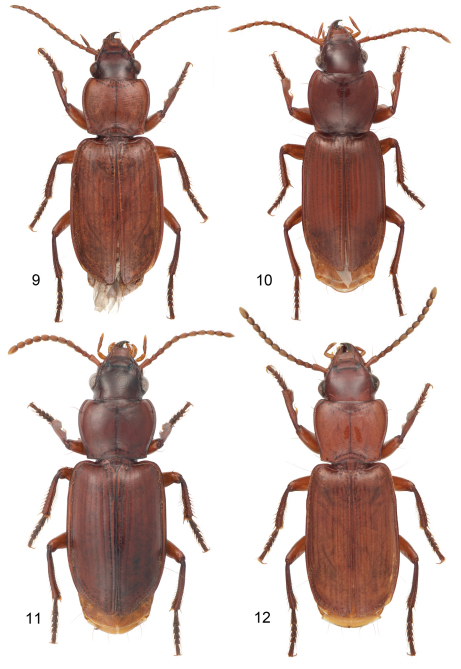
Digital Photo-illustrations, habitus, dorsal aspect: **9** *Mizotrechus fortunensi*s sp. n.**,** ABL = 8.7 mm, male holotype, ADP124968; type locality. **10** *Mizotrechus gorgona*. sp. n.**,** ABL = 8.4 mm, female Holotype, ADP128622; type locality. **11** *Mizotrechus grossus* sp. n.**,** dorsal aspect, ABL = 8.9 mm, male paratype, ADP127169; type locality. **12** *Mizotrechus jefe* sp. n.**,** ABL = 6.6 mm, male paratype, ADP011173; type locality.

#### Dispersal potential.

These beetles are macropterous and capable of flight. They are moderately swift and agile runners.

#### Way of life.

The six adults were collected at UV light trap in premontane forest understory. Adults are active in May, July, and August, in the rainy and early dry seasons.

#### Other specimens examined.

**Paratypes**: Panamá, Chiriqui Province, Fortuna, 1050 m, 8.7341°N, 82.2701°W, 15 May 1978 (H. Wolda)(NMNH: ADP124964, male), 7 July 1978 (H. Wolda)(NMNH: ADP124964, male), 19 July 1978 (H. Wolda)(NMNH: ADP124952, male), 27 July 1977 (H. Wolda)(NMNH: ADP070275, male), 13 August 1978 (H. Wolda)(NMNH: ADP124954, male).

#### Geographic distribution.

 (Fig. 39). This species is currently known only from the type locality in the premontane forested midlands of Panamá.

### Isla Gorgona trough beetle Mizotrechus gorgona sp. n.

urn:lsid:zoobank.org:act:14F5ED42-8D4B-4720-829E-5F1145C10EA9

http://species-id.net/wiki/Mizotrechus_gorgona

Figs 10 24 39

#### Holotype.

COLOMBIA, Cacua, Isla Gorgona Mancora, 60 m, 2.96°N, 78.18°W, 26 June – 18 July 2000 (H Torres)(UASM: ADP128622, female).

#### Derivation of specific epithet.

 The epithet “*gorgona*” is a Latinized noun in apposition and is the name of the island off Colombia where these beetles are found.

#### Proposed English vernacular name.

 Isla Gorgona trough beetle.

#### Diagnosis.

With the attributes of the genus as described above and moderately large sized for the genus as it is presently understood; adults have castaneous integument, except anterior parts of mandible, baso-lateral corner of labrum, and clypeal suture piceous. Frons shallowly rugose above and behind eye in an arc, not extended on occiput, and moderately punctulate. Occiput moderately punctulate. Pronotum longer than wide with lateral margins narrowly explanate and basally with sharp, not denticulate, hind angles; base smooth, without regular microrugose. Elytra moderately narrow and elongate, apex prolonged, wider than the width of pronotum across anterior third, and with 7 well-impressed irregularly punctulate interneurs, intervals not convex; margins behind humeri serrate, intervals between microsetae dentate. Foreleg femur with markedly serrate postero-ventral margin.

#### Description.

(Figs 10, 24). *Size*: See Appendix 1. Moderately large for genus, ABL = 8.4 mm, SBL = 7.15 mm, TW = 2.86 mm. *Color*: see diagnosis, above. *Luster*: Head, pronotum, and legs shiny; elytra matte. *Head*: Labrum quadrate, apico-medially barely emarginate. Eye small and barely convex. Gena straight. Frons, occiput, and gena glabrous. *Prothorax*: Moderately broad, narrowed slightly toward base, margin narrowly explanate, slightly wider before hind angle; surface punctulate, punctures widespread, very fine, glabrous. *Pterothorax*: Elytron barely convex, intervals nearly flat, all interneurs with well-impressed irregularly-spaced punctures, apex prolonged, slightly rounded at extreme sutural apex. *Legs*: Normal in female; foreleg femur (Fig. 24) with markedly serrate postero-ventral margin, not dentate; posterior trochanter tapered to rounded point in female, length half that of femur. *Abdomen*: Abdominal sterna moderately setiferous, densely medially on II and III; sternum VII barely medially notched in female. *Male genitalia*: Unknown. *Female genitalia*: Not investigated; however, it is likely similar to that illustrated on Plate 11.

#### Dispersal potential.

These beetles are macropterous and capable of flight. They are moderately swift and agile runners.

#### Way of life.

The type specimen was collected in a Malaise trap in the rainforest understory at the type locality. Adults are active in late June, or early July during the “relatively” dry season.

#### Other specimens examined.

 None.

#### Geographic distribution.

 (Fig. 39). This species is currently known only from the type locality on an island off the Pacific coast of Colombia.

### Gross trough beetle Mizotrechus grossus sp. n.

urn:lsid:zoobank.org:act:2C262CC2-85AE-4CA2-8BEB-07BEE80B4ADF

http://species-id.net/wiki/Mizotrechus_grossus

Figs 11 32 40 Plate 12

#### Holotype.

GUYANE, Saut Pararé, Arataie River, Nouragues Field Station, 51 m, 4.0378°N, 52.6725°W, 27 March 2010 (S Brule, PH Dalens, & E Poirier)(NMNH: ADP126301, male).

#### Derivation of specific epithet

. The epithet “*grossus*” is a Latin adjective that adequately describes this species with large robust adults.

#### Proposed English vernacular name.

 Gross trough beetle.

#### Diagnosis.

With the attributes of the genus as described above and very large sized for the genus as it is presently understood; adults have dark castaneous integument, except anterior parts of mandible, baso-lateral corner of labrum, and clypeal suture piceous. Frons shallowly rugose at sides and overall punctulate. Occiput punctulate. Pronotum cordiform with lateral margins straight to well-developed hind angle which is dentate; base densely microrugose. Elytra broad and short, moderately wider than the width of pronotum across anterior third, and with 3 well- impressed interneurs, outer interneurs more shallow toward margin; margins behind humeri uneven, but not evidently serrulate. Foreleg femur dentate on postero-ventral margin near base.

#### Description.

(Figs 11, 32, Plate 12). *Size*: See Appendix 1. Large for genus, ABL = 8.1–8.9 mm, SBL = 7.07–7.71 mm, TW = 2.72–3.26 mm. *Color*: see diagnosis, above. *Luster*: Head, pronotum and legs shiny; elytron shiny but with evident shallow and slightly stretched microsculpture. *Head*: Labrum quadrate and medially emarginate. Eye moderately large, moderately convex. Gena moderately long, straight. Frons, occiput, and gena glabrous. *Prothorax*: Broadly cordiform, narrowed moderately toward base, margin narrowly explanate, more broadly near base, hind angle dentate; surface punctulate, punctures widespread, glabrous, microsculpture nearly effaced. *Pterothorax*: Elytron moderately convex, intervals flat, interneurs striate, not punctate, apex oblique and straight, sutural apex narrowly rounded. Metasternum sparsely setiferous in male. *Legs*: Normal in both sexes; foreleg femur (as in Fig. 20) markedly dentate on postero-ventral margin at basal third; trochanter acute in both sexes, about half the length of the femur. *Abdomen*: Abdominal sterna moderately setiferous; sternum IV of male with narrow and dense patch of decumbent setae. Defense gland system (Plate 12) with two very large, medially slightly concave, bean-shaped pygidial gland reservoirs, each with a markedly long and narrow annulated collecting canal connected to the efferent duct near the base of the gland. *Male genitalia*: Median lobe (Fig. 32) elongate and robust with ostium moderately elongate, over half the length of the median lobe; apex with a losp. n.tulate distal end slightly more bent ventrad than in *Mizotrechus poirieri*, moderately curved in lateral aspect, ventral margin proximal to apex straight then evenly curved to apex; endophallus with complexly folded tracheal fields; phallobase hooded and slightly crested, opening more or less 40 degrees off axis of shaft. Parameres large, left a third longer than the right, both broadly rounded, asetose. *Female genitalia*: Not investigated; however, it is likely similar to that illustrated on Plate 11.

#### Dispersal potential.

These beetles are macropterous and capable of flight. They are moderately swift and agile runners.

#### Way of life.

The type series was collected in a flight intercept trap in the rainforest understory at four locations in Guyane. Adults are active in March, April, June, August, October, November, and December, during both the rainy and dry seasons.

#### Other specimens examined. Paratypes.

 Guyane, Saut Pararé, Arataie River, Nouragues Field Station, 51 m, 4.0378°N, 52.6725°W, 26 April 2010 (S Brule, PH Dalens, & E Poirier)(NMNH: ADP127064, female), 15 June 2010 (S Brule, PH Dalens, & E Poirier)(NMNH: ADP127062, ADP127062, females), 24 August 2009 (S Brule, PH Dalens, & E Poirier)(NMNH: ADP124892, female), 10 October 2009 (S Brule, PH Dalens, & E Poirier)(NMNH: ADP124898, male), 30 November 2009 (S Brule, PH Dalens, & E Poirier)(NMNH: ADP124900, female); Mount Itoupe, 570 m, 3.0148°N, 53.0721°W, 17 March 2010 (S Brule, PH Dalens, & E Poirier)(NMNH: ADP126484, 126486, males); Montagne des Chevaux, Commune de Roura, RN2 PK22, 90 m, 4.7127°N, 52.3966°W, 9 August 2009 (S Brule, PH Dalens, & E Poirier)(NMNH: ADP124958, male), 13 December 2009 (S Brule, PH Dalens, & E Poirier)(NMNH: ADP124962, male); Reserve Tresor, Commune de Roura, Route de KAW km 18, 250 m, 4.6104°N, 52.2790°W, 21 November 2009 (S Brule, PH Dalens, & E Poirier)(NMNH: ADP124972, male), Saül, Commune de Saül, Belvédère de Saül, 283–325 m, 3.6223°N, 53.2159°W, 17 November 2010 (S Brule, PH Dalens, & E Poirier)(NMNH: ADP127169, male), Commune de Regina, Reserve Naturelle des Nouragues Inselberg, Petit Plateau, 144 m, 4.0833°N, 52.6833°W, 5 June 2010 (S Brule, PH Dalens, & E Poirier)(NMNH: ADP127157, male), 27 October 2010 (NMNH: ADP127165, male).

**Geographic distribution**. (Fig. 40). This species is currently known from six localities in the lowlands and midlands of Guyane.

### Jefe trough beetle Mizotrechus jefe sp. n.

urn:lsid:zoobank.org:act:3CE7E44F-2EBC-4B71-8251-F041B37D6081

http://species-id.net/wiki/Mizotrechus_jefe

Figs 12 33 39

#### Holotype.

PANAMÁ, Panamá Province, Cerro Jefe, 700–750 m, 9.2311°N, 79.3496°W, 20 May 1972 (RT Allen)(NMNH: ADP011173, female).

#### Derivation of specific epithet.

 The epithet “*jefe*” is a place name, based on the area where the type specimen was collected.

#### Proposed English vernacular name.

 Jefe trough beetle.

#### Diagnosis.

With the attributes of the genus as described above and moderately small sized for the genus as it is presently understood; adults have castaneous integument, except anterior parts of mandible, baso-lateral corner of labrum, and clypeal suture piceous. Frons with rugae nearly effaced, punctulate. Occiput punctulate. Pronotum narrowly cordiform with lateral margins shallowly emarginated; base rugulose. Elytra narrow and short, slightly wider than the width of pronotum across anterior third, and with 8 well-impressed interneurs; margins behind humeri moderately serrulate. Foreleg femur with slightly produced ridge on postero-ventral margin.

#### Description.

(Fig. 12). *Size*: See Appendix 1. Moderately small for genus, ABL = 6.6 mm, SBL = 5.84 mm, TW = 2.27 mm. *Color*: see diagnosis, above. *Luster*: Head, pronotum and legs shiny; elytra shiny yet with evident isodiametric microsculpture. *Head*: Labrum quadrate and medio- apically slightly emarginate. Eye moderately large, moderately convex. Gena moderately long, straight. Frons, occiput, and gena glabrous. *Prothorax*: Moderately narrow, narrowed slightly toward base, margin narrowly explanate throughout, hind angle about right, not dentate; surface punctulate, punctures widespread, glabrous. *Pterothorax*: Elytron slightly convex, disk flat, intervals flat, interneurs striate, not punctate, apex moderately oblique and straight, sutural apex narrowly rounded. Metasternum sparsely setiferous in female. *Legs*: Normal in female; foreleg femur (as in Fig. 21) with slightly produced, short, slightly arcuate ridge on postero-ventral margin at basal forth, not dentate; trochanter narrowly rounded. *Abdomen*: Abdominal sterna moderately setiferous; sternum IV of female with median patch of setae that is more dense than elsewhere. *Male genitalia*: Unknown. *Female genitalia*: Not investigated; however, it is likely similar to that illustrated on Plate 11.

#### Dispersal potential.

These beetles are macropterous and capable of flight. They are moderately swift and agile runners.

#### Way of life.

The adult holotype was collected along a road in the cloud forest understory. Adults are active in May, during the rainy season.

**Other specimens examined**. None.

#### Geographic distribution.

 (Fig. 39). This species is currently known only from the type locality in the midlands of Panamá.

### Laforêt’s trough beetle Mizotrechus marielaforetae sp. n.

urn:lsid:zoobank.org:act:552D9468-9A77-49AF-A12D-5774041E1875

http://species-id.net/wiki/Mizotrechus_marielaforetae

Figs 13 30 40

#### Holotype.

 GUYANE, Montagne des Chevaux, Commune de Roura, RN2 PK22, 90 m, 4.7127°N, 52.3966°W, 28 November 2010 (S Brule, PH Dalens, & E Poirier)(NMNH: ADP127183, male).

#### Derivation of specific epithet.

 The epithet “*marielaforetae*” is an eponym, based on the full stage name of Marie Laforêt, the famous French actress and singer, 5 October 1939 – present, whose voice has a range of diversity like that found in the carabid species richness of Guyane, and who sang a variety of “torch songs”, and here I play on the word “torch”, the same word that applies to that being used to ignite the forests of the unique tropical rainforests of South America, an Armageddon in our own times.

#### Proposed English vernacular name.

 Laforêt’s trough beetle.

#### Diagnosis.

With the attributes of the genus as described above and medium sized for the genus as it is presently understood; adults have castaneous integument, except anterior parts of mandible, baso-lateral corner of labrum, and clypeal suture piceous. Frons without evident rugae, punctulate. Occiput at sides with evident rugae, punctulate. Pronotum nearly quadrate, quite narrowed behind, with lateral margins straight to hind angle, hind angle dentate, tooth small; base densely punctate. Elytra moderately broad and short, slightly wider than the width of pronotum across anterior third, and with only interneur 1 deeply engraved, 2–5 readily evident yet more shallowly impressed, 6–8 traceable, none punctate; margins behind humeri entire, slightly emarginate. Foreleg femur with slightly produced ridge on postero-ventral margin.

#### Description.

(Figs 13, 30). *Size*: See Appendix 1. Moderately small sized for the genus, ABL = 5.9 mm, SBL = 5.12 mm, TW = 2.18 mm. *Color*: see diagnosis, above. *Luster*: Head, pronotum, and legs shiny, elytra duller due to shallowly engraved slightly stretched microsculpture. *Head*: Labrum quadrate and apico-medially emarginate. Eye large, moderately convex. Gena moderately long, straight. Frons, occiput, and gena glabrous. *Prothorax*: Narrow, quadrate, narrowed moderately toward base, margins straight before hind angle, angle dentate, tooth small, margin moderately explanate except wider at hind angle; surface punctulate, punctures widespread, glabrous. *Pterothorax*: Elytron moderately convex, intervals flat, proximal interneurs not punctate, distal interneurs somewhat punctate, apex moderately oblique and straight, sutural apex narrowly rounded. Metasternum sparsely setiferous in male. *Legs*: Normal in male; foreleg femur (as in Fig. 23) with slightly produced ridge on postero-ventral margin, not dentate; posterior trochanter narrowly rounded at apex, about half the length of the femur. *Abdomen*: Abdominal sterna moderately setiferous; sternum IV of male with narrow and dense patch of decumbent setae. *Male genitalia*: Median lobe (Fig. 30) short and robust with ostium moderately elongate, over half the length of the median lobe; apex with a short narrowly rounded distal end, ventral margin proximal to apex straight, then abruptly curved to apex; endophallus with complexly folded tracheal fields; phallobase hooded, opening more or less 40 degrees off axis of shaft. Parameres large, left a third longer than the right, both broadly rounded, asetose. *Female genitalia*: Unknown.

**Plate 4.**
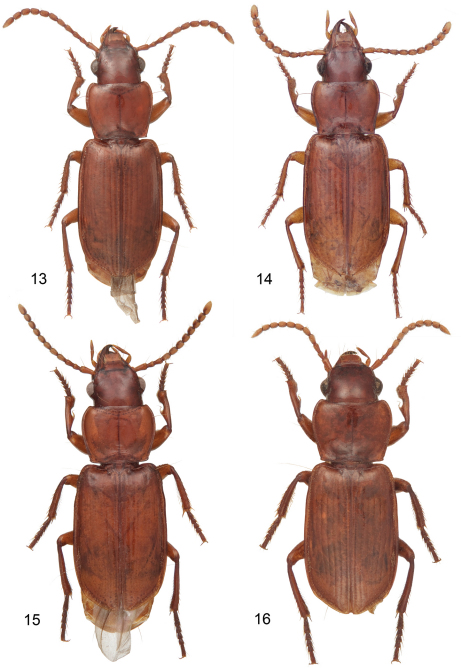
Digital Photo-illustrations, habitus, dorsal aspect: **13** *Mizotrechus marielaforetae* sp. n.**,** dorsal aspect, ABL = 5.9 mm, male paratype, ADP127183, type locality. **14** *Mizotrechus minutus* sp. n.,ABL = 4.4 mm, female paratype, ADP124920; Guyane, Commune de Saül, Belvédère de Saül. **15** *Mizotrechus neblinensis* sp. n.,dorsal aspect, ABL = 6.9 mm, female holotype, ADP124944; type locality. **16** *Mizotrechus novemstriatus* Bates 1872, ABL = 7.2 mm, female lectotype, ADP127163; type locality.

#### Dispersal potential.

These beetles are macropterous and capable of flight. They are moderately swift and agile runners.

#### Way of life.

The adult holotype was collected in a flight intercept trap in the rainforest understory. Adults are active in November, at the end of the dry season.

#### Other specimens examined.

 None.

**Geographic distribution**. (Fig. 40). This species is currently known only from the type locality in the lowlands of Guyane.

### Minute trough beetle Mizotrechus minutus sp. n.

urn:lsid:zoobank.org:act:90504CC6-C4AD-4077-98F4-D5E501F72EB7

http://species-id.net/wiki/Mizotrechus_minutus

Figs 14 19 34 40

#### Holotype.

 GUYANE, Saül, Commune de Saül, Belvédère de Saül, 283–325 m, 3.6223°N, 53.2159°W, 10 December 2010 (S Brule, PH Dalens, & E Poirier)(NMNH: ADP124966, male).

#### Derivation of specific epithet.

 The epithet “*minutus*” is a Latin adjective that adequately describes this species with very small adults.

#### Proposed English vernacular name.

 Minute trough beetle.

#### Diagnosis.

With the attributes of the genus as described above and very small sized for the genus as it is presently understood; adults have castaneous integument, except anterior parts of mandible, baso-lateral corner of labrum, and clypeal suture piceous. Frons with rugae nearly effaced, punctulate. Occiput punctulate. Pronotum quadrate with lateral margins shallowly emarginated; base nearly smooth. Elytra broad and short, slightly wider than the width of pronotum across anterior third, and with 8 well-impressed punctate interneurs; margins behind humeri not serrulate.

#### Description.

(Figs 14, 19, 34). *Size*: See Appendix 1. Very small, ABL = 4.4–4.6 mm, SBL = 3.77–3.88 mm, TW = 1.49–1.58 mm. *Color*: see diagnosis, above. *Luster*: Head, pronotum, elytra, and legs shiny; microsculpture effaced. *Head*: Labrum quadrate and apico-medially emarginate. Eye moderately large, moderately convex. Gena moderately long, straight. Frons, occiput, and genae glabrous. *Prothorax*: Broad, narrowed slightly toward base, margin slightly explanate to hind angle, angle about right, not dentate; surface punctulate, punctures widespread, glabrous. *Pterothorax*: Elytron moderately convex, intervals slightly convex, interneurs striato- punctate, apex moderately oblique and straight, sutural apex narrowly rounded. Metasternum sparsely setiferous in male. *Legs*: Normal in both sexes; foreleg femur unmodified (Fig. 19); trochanter narrowly rounded. *Abdomen*: Abdominal sterna moderately setiferous; sternum IV of male with narrow and dense patch of decumbent setae. *Male genitalia*: Median lobe (Fig. 34) elongate and narrow with ostium moderately elongate, over half the length of the median lobe; apex with a long narrow distal end, straight in lateral aspect and narrowly rounded, ventral margin proximal to apex straight; endophallus with complexly folded tracheal fields; phallobase hooded, opening more or less 15 degrees off axis of shaft. Parameres large, left a third longer than the right, both broadly rounded, asetose. *Female genitalia*: Not investigated; however, it is likely similar to that illustrated on Plate 11.

#### Dispersal potential.

These beetles are macropterous and capable of flight. They are moderately swift and agile runners.

#### Way of life.

The adult holotype was collected in a flight intercept trap in the rainforest understory. Adults are active in December, at the end of the dry season.

#### Other specimens examined.

**Paratypes**: Guyane, Saut Pararé, Arataie River, Nouragues Field Station, 51 m, 4.0378°N, 52.6725°W, 11 December 2009 (S Brule, PH Dalens, & E Poirier)(NMNH: ADP124902, male), Saül, Commune de Saül, Belvédère de Saül, 283–325 m, 3.6223°N, 53.2159°W, 10 December 2010 (S Brule, PH Dalens, & E Poirier)(NMNH: ADP124920, female).

#### Geographic distribution.

 (Fig. 40). This species is currently known only from two localities in the lowlands of Guyane.

### Tipui trough beetle Mizotrechus neblinensis sp. n.

urn:lsid:zoobank.org:act:11B2AD1D-46FC-45BD-8F93-4BA20D3CA2CF

http://species-id.net/wiki/Mizotrechus_neblinensis

Figs 15 35 40 41

#### Holotype.

VENEZUELA**,** Amazonas, Cerro de la Neblina (Smithsonian Basecamp), 140 m, 0.0148°N, 66.1604°W, 10–20 February 1985 (PJ Spangler, PM Spangler, RE Faitoute & WE Steiner)(NMNH: ADP124944, female).

#### Derivation of specific epithet.

 The epithet “***neblinensis***” is a Latinized noun in apposition, based on a geographic name formed from Pico de Neblina, a tipui on the border of Venezuela and Brazil, the area in which adults of this species have been found, and the Latin suffix „-*ensis*,“ meaning of, or from that place.

#### Proposed English vernacular name.

 Tipui trough beetle.

#### Diagnosis.

With the attributes of the genus as described above and medium sized for the genus as it is presently understood; adults have castaneous integument, except anterior parts of mandible, baso-lateral corner of labrum, and clypeal suture piceous. Frons with nearly effaced rugae, punctulate. Occiput punctulate. Pronotum nearly quadrate with lateral margins straight to hind angle, hind angle a very small tooth; base densely rugulose. Elytra broad and short, slightly wider than the width of pronotum across anterior third, and with 6 evident shallowly impressed interneurs, 7–8 nearly effaced; margins behind humeri moderately serrulate. Foreleg femur with markedly developed and produced ridge on postero-ventral margin.

#### Description.

(Figs 15, 35). *Size*: See Appendix 1. Medium sized for the genus, ABL = 6.4–6.9 mm, SBL = 5.54–6.12 mm, TW = 2.25–2.46 mm. *Color*: see diagnosis, above. *Luster*: Head, pronotum, elytra and legs shiny, microsculpture absent. *Head*: Labrum quadrate and apico- medially emarginate. Eye large, moderately convex. Gena moderately long, straight. Frons, occiput, and gena glabrous. *Prothorax*: Broad, narrowed slightly toward base, margin narrowly explanate except wider at hind angle, hind angle a very small tooth; surface punctulate, punctures widespread, glabrous. *Pterothorax*: Elytron moderately convex, disk flat, intervals flat, interneurs striato-punctate, punctures small, shallow, apex oblique and straight, sutural apex narrowly rounded. Metasternum sparsely setiferous in male. *Legs*: Normal in both sexes; foreleg femur (as in Fig. 22) ridged along the posterio-ventral margin and markedly produced at middle of ridge; posterior trochanter narrowly rounded at apex, about half the length of the femur. *Abdomen*: Abdominal sterna moderately setiferous; sternum IV of female with denser patch of setae at midline. *Male genitalia*: Median lobe (Fig. 35) short and robust with ostium moderately elongate, over half the length of the median lobe; apex a short blunt distal end, slightly curved in lateral aspect, ventral margin proximal to apex evenly arched and barely curved to apex; endophallus with complexly folded tracheal fields; phallobase hooded, opening more or less five degrees off axis of shaft. Parameres large, left a third longer than the right, both broadly rounded, asetose. *Female genitalia*: Not investigated; however, it is likely similar to that illustrated on Plate 11.

#### Dispersal potential.

These beetles are macropterous and capable of flight. They are moderately swift and agile runners.

#### Way of life.

The adult holotype was collected in a flight intercept trap in the rainforest understory. Adults are active in February, in the rainy season.

#### Other specimens examined.

**Paratypes**: Guyane, Montagne des Chevaux, Commune de Roura, RN2 PK22, 90 m, 4.7127°N, 52.3966°W, 19 September 2009 (NMNH: ADP124970, male), 31 October 2010 (NMNH: ADP128733, male). Venezuela**,** Amazonas, Cerro de la Neblina (Smithsonian Basecamp), 140 m, 0.0148°N, 66.1604°W, 10–20 February 1985 (PJ Spangler, PM Spangler, RE Faitoute & WE Steiner)(NMNH: ADP124948, female).

#### Geographic distribution

. (Figs 40, 41). This species is currently known from lowland localities, one each in Guyane and Venezuela.

### Nine-lined trough beetle Mizotrechus novemstriatus

Bates, 1872

http://species-id.net/wiki/Mizotrechus_novemstriatus

Figs 16 41

#### Lectotype.

**(**labeled by me in Paris, early 1970’s**)**: BRAZIL: Amazonas, Ega (Tefé) approximately 3.35°S, 64.71°W, 46 m (HW Bates)(MNHP: female). ADP127163.

#### Derivation of specific epithet.

 The epithet “*novemstriatus*” is descriptive Latin, meaning 9-lined and referring to the elytral interneurs, including the scutellar striole.

#### Proposed English vernacular name.

 Nine-lined trough beetle.

#### Diagnosis.

With the attributes of the genus as described above and moderate sized for the genus as it is presently understood; adults have castaneous integument, except anterior parts of mandible, baso-lateral corner of labrum, and clypeal suture piceous. Frons smooth, scattered rugulae not organized, moderately punctulate. Occiput smooth, moderately punctulate. Pronotum subcordiform with lateral margins narrowly explanate and basally with sharp, not denticulate, hind angles; base with regular rugulae. Elytra broad and short, wider than the width of pronotum across anterior third, and with 8 well-impressed irregularly punctulate interneurs, intervals not convex, apex not prolonged, margins behind humeri rough, intervals between microsetae blunt. Foreleg femur with evident ridge on postero-ventral margin.

#### Description.

(Fig. 16). *Size*: See Appendix 1. Moderate sized for genus, ABL = 7.2 mm, SBL = 6.05 mm, TW = 2.48 mm. *Color*: see diagnosis, above. *Luster*: Head, pronotum, elytra, and legs shiny. *Head*: Labrum quadrate, apico-medially v-notched. Eye small and moderately convex. Gena straight. Frons, occiput, and gena glabrous. *Prothorax*: Moderately broad, narrowed slightly toward base, margin narrowly explanate, slightly wider before hind angle; surface punctulate, punctures widespread, very fine, glabrous. *Pterothorax*: Elytron barely convex, intervals nearly flat, all interneurs with well-impressed irregularly-spaced punctulate, apex not prolonged, slightly rounded at extreme sutural apex. *Legs*: Normal in female; foreleg femur (as in Fig. 22) ridged along the postero-ventral margin and markedly produced at middle of ridge; posterior trochanter tapered to rounded point in female, length half that of femur. *Abdomen*: Abdominal sterna moderately setiferous, densely medially on II and III; sternum VII barely medially notched in female. *Male genitalia*: Unknown. *Female genitalia*: Not investigated; however, it is likely similar to that illustrated on Plate 11.

**Plate 5.**
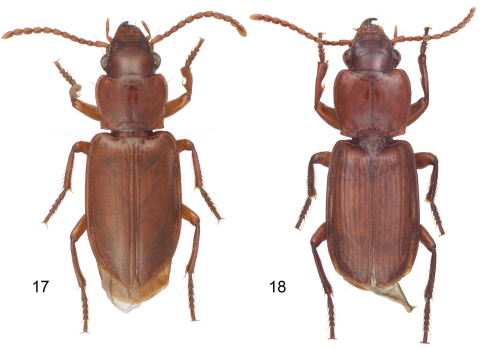
Digital Photo-illustrations, habitus, dorsal aspect: **17** *Mizotrechus poirieri* sp. n., ABL = 6.6 mm, male holotype, ADP124888; type locality. **18** *Mizotrechus woldai* sp. n., dorsal aspect, ABL = 7.4 mm, female holotype, ADP124946; type locality.

#### Dispersal potential.

These beetles are macropterous and capable of flight. They are moderately swift and agile runners.

#### Way of life.

Unknown.

#### Other specimens examined.

 None.

#### Geographic distribution.

 (Fig. 41). This species is currently known only from the type locality in the lowlands of Brazil.

### Poirier's trough beetle Mizotrechus poirieri sp. n.

urn:lsid:zoobank.org:act:FA89B987-1B34-4AAA-B5A4-DE4971821A76

http://species-id.net/wiki/Mizotrechus_poirieri

Figs 17 36 40

#### Holotype.

GUYANE, Saut Pararé, Arataie River, Nouragues Field Station, 51 m, 4.0378°N, 52.6725°W, 30 November 2009 (S Brule, PH Dalens, & E Poirier)(NMNH: ADP124888, male).

#### Derivation of specific epithet.

 The epithet “*poirieri*” is an eponym, based on the family name of E. Poirieri, whose team in Guyane has been collecting beetles using Flight Intercept Traps and capturing adults of many new species, such as this one.

#### Proposed English vernacular name.

 Poirier’s trough beetle.

#### Diagnosis.

With the attributes of the genus as described above and medium sized for the genus as it is presently understood; adults have castaneous integument, except anterior parts of mandible, baso-lateral corner of labrum, and clypeal suture piceous. Frons with evident rugae, punctulate. Occiput with evident rugae, punctulate. Pronotum nearly quadrate with lateral margins slightly emarginate to hind angle, hind angle about right, not dentate; base sparsely rugulose. Elytra broad and short, much wider than the width of pronotum across anterior third, and with only interneur 1 deeply engraved, 2–8 evident yet shallowly impressed, not punctate; margins behind humeri entire. Foreleg femur with slightly produced swelling on postero-ventral margin.

#### Description.

(Figs 17, 36). *Size*: See Appendix 1. Medium sized for the genus, ABL = 6.6 mm, SBL = 5.65 mm, TW = 2.42 mm. *Color*: see diagnosis, above. *Luster*: Head, pronotum, and legs shiny, elytra duller due to shallowly engraved slightly stretched microsculpture. *Head*: Labrum quadrate and apico-medially emarginate. Eye large, moderately convex. Gena moderately long, straight. Frons, occiput, and gena glabrous. *Prothorax*: Narrow, quadrate, narrowed slightly toward base, margins slightly emarginated before hind angle, angle about right, not dentate, margin moderately explanate except wider at hind angle; surface punctulate, punctures widespread, glabrous. *Pterothorax*: Elytron moderately convex, disk flat, intervals flat, interneurs not punctate, apex markedly oblique and straight, sutural apex narrowly rounded. Metasternum sparsely setiferous in male. *Legs*: Normal in male; foreleg femur (as in Fig. 20) with a minute setose tooth on postero-ventral margin, subdentate; posterior trochanter narrowly acute at apex, about half the length of the femur. *Abdomen*: Abdominal sterna moderately setiferous; sternum IV of male with narrow and dense patch of decumbent setae. *Male genitalia*: Median lobe (Fig. 36) elongate and robust with ostium moderately elongate, over half the length of the median lobe; apex a losp. n.tulate distal end that is more bent ventrad than in *Mizotrechus dalensi*, moderately curved in lateral aspect, ventral margin proximal to apex straight then evenly curved to apex; endophallus with complexly folded tracheal fields; phallobase hooded and crested, opening more or less 20 degrees off axis of shaft. Parameres large, left a third longer than the right, both broadly rounded, asetose. *Female genitalia*: Unknown.

#### Dispersal potential.

These beetles are macropterous and capable of flight. They are moderately swift and agile runners.

#### Way of life.

The adult holotype was collected in a flight intercept trap in the rainforest understory. Adults are active in November, at the end of the dry season.

#### Other specimens examined.

 None.

#### Geographic distribution.

 (Fig. 40). This species is currently known only from the type locality in the lowlands of Guyane.

### Wolda’s trough beetle Mizotrechus woldai sp. n.

urn:lsid:zoobank.org:act:2806BBDA-1FDF-4030-B08F-4BAF13DBBC60

http://species-id.net/wiki/Mizotrechus_woldai

Figs 18 39

#### Holotype.

PANAMÁ, Canal Zone, Barro Colorado Island, 99 m, 9.1628°N, 79.8395°W, 5 May 1978 (H Wolda)(NMNH: ADP 124946, female).

#### Derivation of specific epithet.

 The epithet “*woldai*” is an eponym, based on the family name of H. Wolda, long time scientist with the Smithsonian Tropical Research Institute in Panamá, whose persistent light trapping produced many new and interesting species, such as one adult of this new species.

#### Proposed English vernacular name.

 Wolda’s trough beetle.

#### Diagnosis.

With the attributes of the genus as described above and medium sized for the genus as it is presently understood; adults have castaneous integument, except anterior parts of mandible, baso-lateral corner of labrum, and clypeal suture piceous. Frons with evident rugae, punctulate. Occiput without rugae, punctulate. Pronotum nearly quadrate with lateral margins slightly emarginate to hind angle, hind angle dentate, tooth small; base sparsely rugulose. Elytra broad and short, slightly wider than the width of pronotum across anterior third, and with only interneurs 8 deeply engraved, not punctate; margins behind humeri serrate.

#### Description.

(Fig. 18). *Size*: See Appendix 1. Medium sized for the genus, ABL = 7.4 mm, SBL = 6.04 mm, TW = 2.29 mm. *Color*: see diagnosis, above. *Luster*: Head, pronotum, and legs shiny, elytra duller due to shallowly engraved slightly stretched microsculpture. *Head*: Labrum quadrate and apico-medially emarginate. Eye large, moderately convex. Gena moderately short, straight. Frons, occiput, and gena glabrous. *Prothorax*: Very broad, quadrate, narrowed slightly toward base, margins slightly emarginated before hind angle, angle dentate, margin beaded, moderately explanate at hind angle; surface punctulate, punctures widespread, glabrous. *Pterothorax*: Elytron flat, disk flat, intervals flat, interneurs not punctate, apex moderately oblique and straight, sutural apex narrowly rounded. Metasternum sparsely setiferous in male. *Legs*: Normal in female; foreleg femur (as in Fig. 21) with slightly produced, short, arcuate ridge on postero-ventral margin at basal forth, not dentate. *Abdomen*: Abdominal sterna moderately setiferous. *Male genitalia*: Unknown. *Female genitalia*: Not investigated; however, it is likely similar to that illustrated on Plate 11.

**Plate 6.**
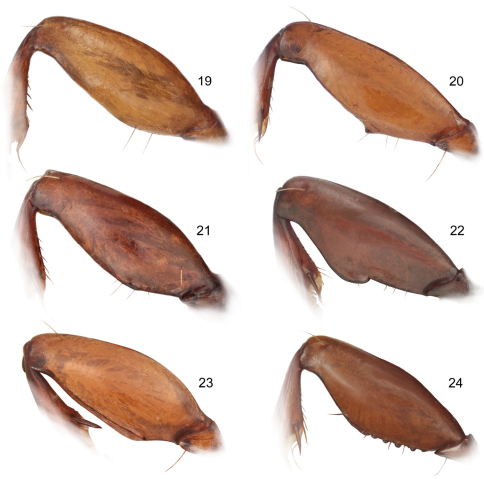
Digital Photo-illustrations, anterior femur, ventral aspect: **19** *Mizotrechus minutus* sp. n., ADP124966; type locality. **20** *Mizotrechus dalensi* sp. n., ADP124896; type locality. **21** *Mizotrechus chontalesensis* sp. n., ADP127181; type locality. **22** *Mizotrechus edithpiafae* sp. n., ADP124948; type locality. **23** *Mizotrechus brulei* sp. n., ADP129205; type locality. **24** *Mizotrechus gorgona*. sp. n., ADP128622; type locality.

#### Dispersal potential.

These beetles are macropterous and capable of flight. They are moderately swift and agile runners.

#### Way of life.

The adult holotype was collected in a ground level UV light trap in the rainforest understory. Adults are active in May during the heavy rainy season in Panamá.

#### Other specimens examined.

None.

#### Geographic distribution.

 (Fig. 39). This species is currently known from a single lowland locality in Panamá.

#### Note.

Considering the hundreds of specimens of carabids collected by Wolda in a series of suspended light traps from canopy level to ground level, it is curious that no other specimens of this species were collected (see *Mizotrechus fortunensis* above, where many specimens were collected by Wolda at light traps).

**Plate 7.**
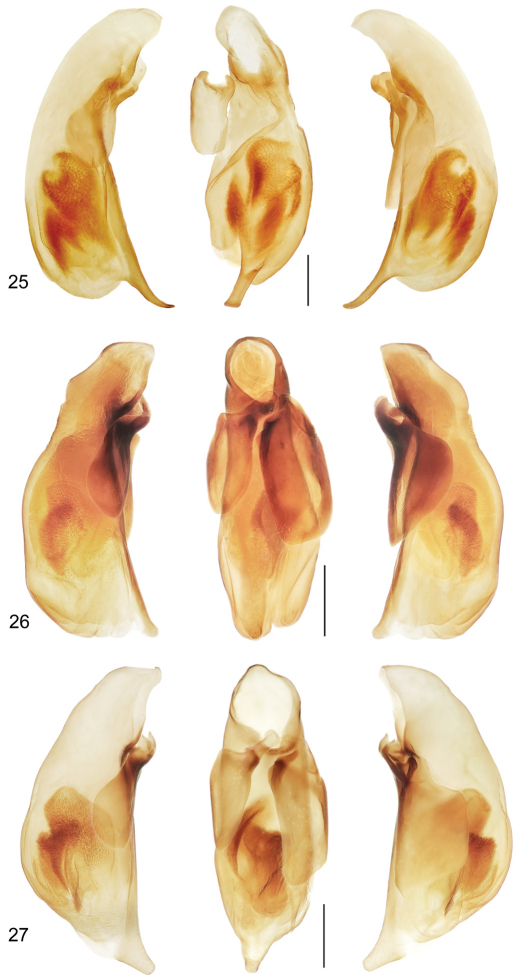
Digital Photo-illustrations, male genitalia, median lobe and parameres, left lateral, dorsal, and right lateral aspects: **25** *Mizotrechus bellorum* sp. n., ADP124890; type locality. **26** *Mizotrechus belvedere* sp. n., ADP129201; type locality. **27** *Mizotrechus brulei* sp. n., ADP127159, Guyane, Montagne des Chevaux, Commune de Roura.

**Plate 8.**
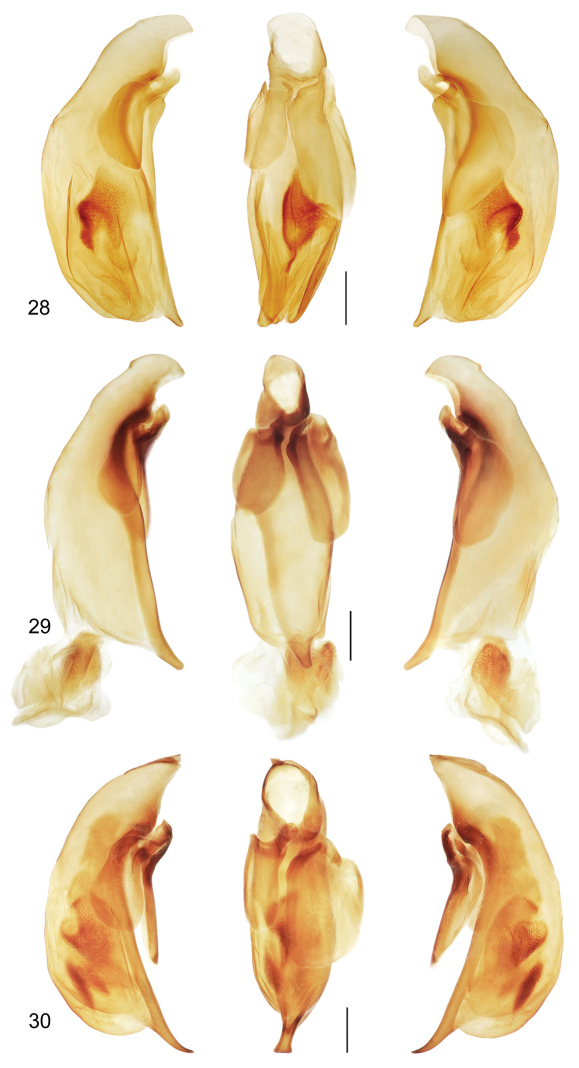
Digital Photo-illustration, male genitalia, median lobe and parameres, left lateral, dorsal, and right lateral aspects: **28** *Mizotrechus costaricensis* sp. n., ADP128620: type locality. **29** *Mizotrechus dalensi* sp. n., ADP129203, Guyane, Saut Pararé, Arataie River, Nouragues Field Station. **30** *Mizotrechus edithpiafae* sp. n., ADP129203; G^er^ ?locality (see text).

**Plate 9.**
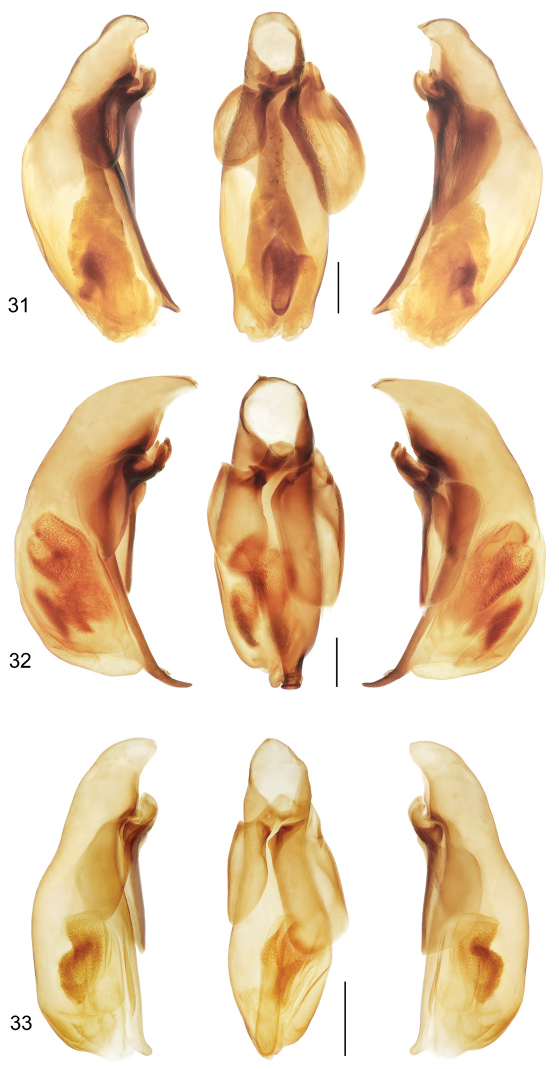
Digital Photo-illustration, male genitalia, median lobe, left lateral, dorsal, and right lateral aspects: **31** *Mizotrechus fortunensis* sp. n., ADP124590; type locality. **32** *Mizotrechus grossus* sp. n., ADP127165; type locality. **33** *Mizotrechus marielaforetae* sp. n., ADP127183; type locality.

**Plate 10.**
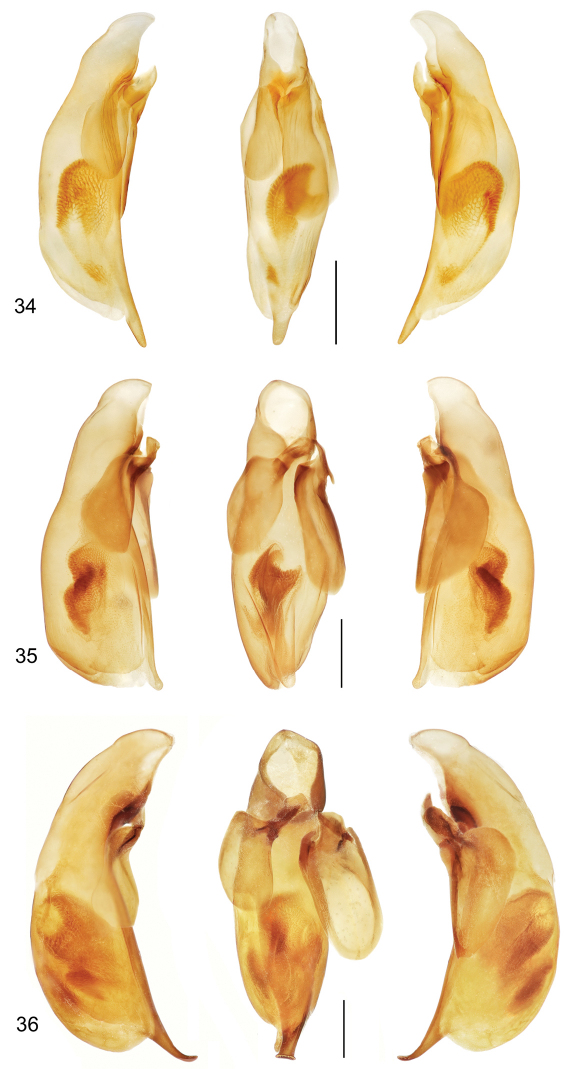
Digital Photo-illustration, male genitalia, median lobe, left lateral, dorsal, and right lateral aspects: **34** *Mizotrechus minutus* sp. n., ADP124902; type locality. **35** *Mizotrechus neblinensis* sp. n., ADP124944; type locality. **36** *Mizotrechus poirieri* sp. n., ADP124888; type locality.

**Plate 11.**
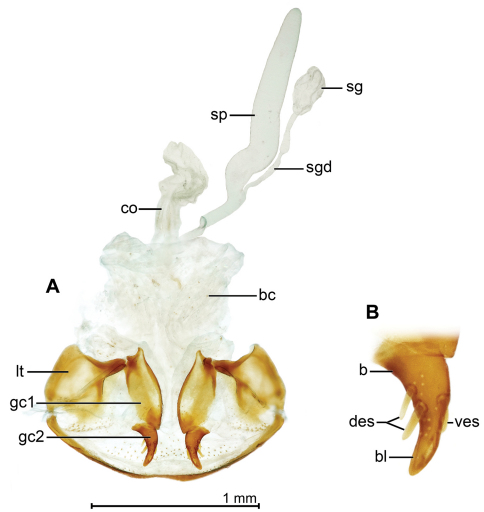
**Figure 37.** Digital Photo-illustration, female genitalia: *Mizotrechus dalensi* sp. n., ADP127161; Petite Montague Tortue, Guyane. A. Dorsal aspect. Legend, **bc,** bursa copulatrix; **co,** common oviduct; **sg,** spermathecal gland; **sgd**, spermathecal gland duct; sp, spermatheca. dorsal aspect; **vc**, villous canal; **lt**, laterotergite; **gc1**, gonocoxite 1; **gc2**, gonocoxite 2. B. Gonocoxite 2, dorsal aspect: Legend, **b,** base of gonocoxite 2; **bl**, blade of gonocoxite 2; **des**, dorsal ensiform seta; **ves,** ventral ensiform setae**.**

**Plate 12.**
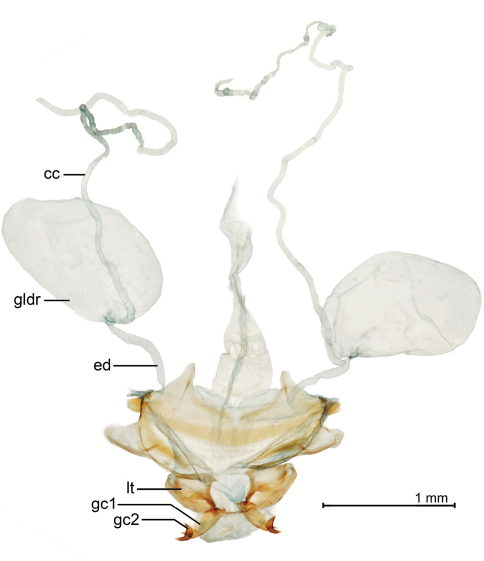
**Figure 38.** Digital Photo-illustration, pygidial (defense) gland system: *Mizotrechus grossus* sp. n., dorsal aspect, ADP127064; type locality. Legend: **cc**, collecting canal; **ed**, efferent duct; **edbl**, efferent duct, basal lobe; **gldr**, pygidial gland reservoir. Ovipositor sclerites, Legend: **gc1,** gonocoxite 1; **gc2,** gonocoxite 2; **lt**, laterotergite.

## Discussion

For 108 years, adults of *Mizotrechus* Bates 1872 have flown “under the radar,” so to speak. Then, along came the FITs (Flight Intercept Traps) used by the SEAG group in Guyane. Since collection of the unique holotype of *Mizotrechus novemstriatus* Bates from Ega (now Tefé), Brazil, and a misidentified (by Bates) specimen collected by Janson in Nicaragua, this genus has remained an enigma and rarely collected. All the very few specimens that have resided in collections have been determined as *Mizotrechus novemstriatus* Bates, because the genus was thought to be monobasic. The synopsis presented in this paper is an opening salvo, only. There is no question that the use of FITs, in the rest of the Oronoco and Amazon Basins and in Middle America, will produce many new species in addition to those described herein. The type locality of the type species is south of the Amazon River; and this suggests that there may be many more species residing on the Brazilian Shield, just as there are on the Guyana Shield, as revealed by this study.

With the increase in our knowledge from two specimens to 56 specimens and from one species to 18 species, this poorly known genus now has become recognized as a likely dominate subcortical (or leaf litter?) inhabitant of the Neotropics. The Tribe Perigonini is little known overall and has never been a group seriously studied in the Western Hemisphere. It is very likely that the other two described genera, *Perigona* Laporte de Castelnau 1835 and *Diploharpus* Chaudoir 1850, and a third as yet undescribed genus (Erwin, in prep) will also be replete with new species, as is *Mizotrechus*, once they are studied. For example, *Diploharpus* is now known to have 13 described species assigned to it throughout its entire range from México south to Bolivia and Brazil. In the Guyane FIT samples alone, I have sorted out 27 morphospecies of *Diploharpus*.

While writing descriptions and creating the key, it became apparent that certain specific attributes have begun to form biogeographic patterns. The serrate humerus is found in the Central America/Colombia species and one Guyane species, and the serrate femur in the same species in Central America/Colombia only; the dentate femora only in the Guyane species. When more species are discovered and documented/described, this genus has the potential of contributing to a better understanding of the biogeography of the Central American/northern South American connections.

As I have documented with my fogging assemblages, a novel collecting technique opens new and exciting faunal components to scientific investigation and knowledge. Unfortunately, the opening of new taxonomic vistas with these new collecting techniques, and discovery of the new and/or rare species found, comes at a time when taxonomists themselves are becoming rare (Pearson et al. 2010; Cardosa et al. 2011). While these authors provide some solutions, the question remains: Will those solutions be enough and in time, while biodiversity is still here, to help us fully grasp what global species richness means (Erwin et al. 2005)?

## Supplementary Material

XML Treatment for Mizotrechus

XML Treatment for Mizotrechus batesi

XML Treatment for Mizotrechus bellorum

XML Treatment for Mizotrechus belvedere

XML Treatment for Mizotrechus brulei

XML Treatment for Mizotrechus chontalesensis

XML Treatment for Mizotrechus costaricensis

XML Treatment for Mizotrechus dalensi

XML Treatment for Mizotrechus edithpiafae

XML Treatment for Mizotrechus fortunensis

XML Treatment for Mizotrechus gorgona

XML Treatment for Mizotrechus grossus

XML Treatment for Mizotrechus jefe

XML Treatment for Mizotrechus marielaforetae

XML Treatment for Mizotrechus minutus

XML Treatment for Mizotrechus neblinensis

XML Treatment for Mizotrechus novemstriatus

XML Treatment for Mizotrechus poirieri

XML Treatment for Mizotrechus woldai

## Figures and Tables

**Table:** Tables with measurements and ratios for adults of species of *Mizotrechus* Bates 1872. All measurements in millimeters. Apparent body length (ABL) provided in the descriptions.

**TOTAL LENGTH (SBL)**						
	**Males**			**Females**		
**Species**	**N**	**Range**	**Mean**	**N**	**Range**	**Mean**
*Mizotrechus batesi*	1	4.897				
*Mizotrechus bellorum*	1	5.874				
*Mizotrechus belvedere*	1	6.142				
*Mizotrechus brulei*	2	5.535–5.644	5.59	7	6.408–7.096	6.759
*Mizotrechus chontalesensis*				1	5.886	
*Mizotrechus costaricensis*	1	6.888				
*Mizotrechus dalensi*	4	6.404–7.101	6.736	4	6.408–7.096	6.759
*Mizotrechus edithpiafae*	1	7.162				
*Mizotrechus fortunensis*	6	7.02–7.66	7.256			
*Mizotrechus gorgona*				1	7.15	
*Mizotrechus grossus*	10	7.068–7.711	7.301	4	6.986–7.643	7.257
*Mizotrechus jefe*				1	5.836	
*Mizotrechus marielaforetae*	1	5.123				
*Mizotrechus minutus*	1	3.767		2	3.7341–3.879	3.807
*Mizotrechus neblenaensis*	2	5.665–5.69	5.678	2	5.539–6.118	5.829
*Mizotrechus novemstriatus*				1	6.045	
*Mizotrechus poirieri*	1	5.649				
*Mizotrechus woldai*				1	6.404	
**MAXIMUM WIDTH**						
	**Males**			**Females**		
**Species**	**N**	**Range**	**Mean**	**N**	**Range**	**Mean**
*Mizotrechus batesi*	1	1.792				
*Mizotrechus bellorum*	1	2.556				
*Mizotrechus belvedere*	1	2.484				
*Mizotrechus brulei*	2	2.036–2.046	2.041	7	1.986–2.224	2.123
*Mizotrechus chontalesensis*				1	2.286	
*Mizotrechus costaricensis*	1	2.888				
*Mizotrechus dalensi*	4	2.646–2.964	2.813	4	2.724–3.114	2.833
*Mizotrechus edithpiafae*	1	2.942				
*Mizotrechus fortunaensis*	6	2.928–3.032	2.972			
*Mizotrechus gorgona*				1	2.86	
*Mizotrechus grossus*	10	2.72–3.114	2.974	4	2.846–3.262	2.994
*Mizotrechus jefe*				1	2.272	
*Mizotrechus marielaforetae*	1	2.178				
*Mizotrechus minutus*	1	1.49		2	1.51–1.576	1.543
*Mizotrechus neblenaensis*	2	2.256–2.358	2.307	2	2.3–2.464	2.382
*Mizotrechus novemstriatus*				1	2.48	
*Mizotrechus poirieri*	1	2.418				
*Mizotrechus woldai*				1	2.286	
**W of HEAD/W of L. ELYTRON**						
	**Males**			**Females**		
**Species**	**N**	**Range**	**Mean**	**N**	**Range**	**Mean**
*Mizotrechus batesi*	1	1.434				
*Mizotrechus bellorum*	1	1.191				
*Mizotrechus belvedere*	1	1.196				
*Mizotrechus brulei*	2	1.459–1.495	1.477	7	1.213–1.434	1.307
*Mizotrechus chontalesensis*				1	1.358	
*Mizotrechus costaricensis*	1	1.139				
*Mizotrechus dalensi*	4	1.199–1.274	1.234	4	1.170–1.287	1.229
*Mizotrechus edithpiafae*	1	1.218				
*Mizotrechus fortunaensis*	6	1.057–1.151	1.109			
*Mizotrechus gorgona*				1	1.192	
*Mizotrechus grossus*	10	1.201–1.324	1.238	4	1.186–1.281	1.227
*Mizotrechus jefe*				1	1.3	
*Mizotrechus marielaforetae*	1	1.198				
*Mizotrechus minutus*	1	1.27		2	1.280–1.282	1.281
*Mizotrechus neblenaensis*	2	1.207–1.238	1.223	2	1.276–1.306	1.291
*Mizotrechus novemstriatus*				1	1.325	
*Mizotrechus poirieri*	1	1.191				
*Mizotrechus woldai*				1	1.358	
**PRONOTUM: WIDTH (at widest part)/LENGTH**						
	**Males**			**Females**		
**Species**	**N**	**Range**	**Mean**	**N**	**Range**	**Mean**
*Mizotrechus batesi*	1	1.179				
*Mizotrechus bellorum*	1	1.283				
*Mizotrechus belvedere*	1	1.208				
*Mizotrechus brulei*	2	1.192–1.236	1.214	7	1.204–1.308	1.248
*Mizotrechus chontalesensis*				1	1.226	
*Mizotrechus costaricensis*	1	1.236				
*Mizotrechus dalensi*	4	1.333–1.362	1.347	4	1.305–1.414	1.362
*Mizotrechus edithpiafae*	1	1.281				
*Mizotrechus fortunaensis*	6	1.179–1.249	1.215			
*Mizotrechus gorgona*				1	1.194	
*Mizotrechus grossus*	10	1.303–1.383	1.344	4	1.295–1.360	1.324
*Mizotrechus jefe*				1	1.206	
*Mizotrechus marielaforetae*	1	1.222				
*Mizotrechus minutus*	1	1.311		2	1.315–1.316	1.315
*Mizotrechus neblenaensis*	2	1.262–1.328	1.295	2	1.281–1.347	1.314
*Mizotrechus novemstriatus*				1	1.356	
*Mizotrechus poirieri*	1	1.308				
*Mizotrechus woldai*				1	1.168	
**LENGTH OF PRONOTUM / LENGTH OF HEAD**						
	**Males**			**Females**		
**Species**	**N**	**Range**	**Mean**	**N**	**Range**	**Mean**
*Mizotrechus batesi*	1	1.938				
*Mizotrechus bellorum*	1	1.897				
*Mizotrechus belvedere*	1	2.29				
*Mizotrechus brulei*	2	2.059–2.121	2.09	7	1.965–2.201	2.073
*Mizotrechus chontalesensis*				1	2.243	
*Mizotrechus costaricensis*	1	2.4				
*Mizotrechus dalensi*	4	2.061–2.231	2.134	4	1.95–2.186	2.06
*Mizotrechus edithpiafae*	1	2.456				
*Mizotrechus fortunaensis*	6	2.144–2.347	2.206			
*Mizotrechus gorgona*				1	2.275	
*Mizotrechus grossus*	10	1.942–2.265	2.08	4	1.938–2.288	2.087
*Mizotrechus jefe*				1	2.075	
*Mizotrechus marielaforetae*	1	2.263				
*Mizotrechus minutus*	1	2.113		2	2.097–2.233	2.165
*Mizotrechus neblenaensis*	2	2.001–2.257	2.129	2	1.996–2.228	2.112
*Mizotrechus novemstriatus*				1	2.188	
*Mizotrechus poirieri*	1	1.892				
*Mizotrechus woldai*				1	2.812	
